# Odd-parity quasiparticle interference in the superconductive surface state of UTe_2_

**DOI:** 10.1038/s41567-025-03000-w

**Published:** 2025-09-18

**Authors:** Shuqiu Wang, Kuanysh Zhussupbekov, Joseph P. Carroll, Bin Hu, Xiaolong Liu, Emile Pangburn, Adeline Crepieux, Catherine Pepin, Christopher Broyles, Sheng Ran, Nicholas P. Butch, Shanta Saha, Johnpierre Paglione, Cristina Bena, J. C. Séamus Davis, Qiangqiang Gu

**Affiliations:** 1 Clarendon Laboratory, University of Oxford, Oxford, UK; 2 H. H. Wills Physics Laboratory, University of Bristol, Bristol, UK; 3 Department of Physics, University College Cork, Cork, Ireland; 4 LASSP, Department of Physics, Cornell University, Ithaca, NY USA; 5 Department of Physics, University of Notre Dame, Notre Dame, IN USA; 6 Institut de Physique Théorique, Université Paris Saclay, CEA CNRS, Gif-sur-Yvette, France; 7 Aix Marseille Univ, Université de Toulon, CNRS, CPT, Marseille, France; 8 Department of Physics, Washington University, St Louis, MO USA; 9 Maryland Quantum Materials Center, University of Maryland, College Park, MD USA; 10 Canadian Institute for Advanced Research, Toronto, Ontario Canada; 11 Max-Planck Institute for Chemical Physics of Solids, Dresden, Germany; 12 Tsung-Dao Lee Institute, Shanghai Jiao Tong University, Shanghai, China

**Keywords:** Superconducting properties and materials, Topological matter

## Abstract

Although no known material exhibits intrinsic topological superconductivity, where a spin-triplet electron pairing potential has odd parity, UTe_2_ is now the leading candidate. Generally, the parity of a superconducting order parameter can be established using Bogoliubov quasiparticle interference imaging. However, odd-parity superconductors should support a topological quasiparticle surface band at energies within the maximum superconducting energy gap. Quasiparticle interference should then be dominated by the electronic structure of the quasiparticle surface band and only reveal the characteristics of the bulk order parameter indirectly. Here we demonstrate that at the (0–11) cleave surface of UTe_2_, a band of Bogoliubov quasiparticles appears only in the superconducting state. Performing high-resolution quasiparticle interference measurements then allows us to explore the dispersion of states in this superconductive surface band, showing that they exist only within the range of Fermi momenta projected onto the (0–11) surface. Finally, we develop a theoretical framework to predict the quasiparticle interference signatures of this surface band at the (0–11) surface. Its predictions are consistent with the experimental results if the bulk superconducting order parameter exhibits time-reversal conserving, odd-parity, *a*-axis nodal, *B*_3__*u*_ symmetry.

## Abstract (web summary)

UTe_2_ is a proposed intrinsic topological superconductor, but its quasiparticle surface band has not yet been visualized. Now this is achieved using quasiparticle interference imaging, revealing the symmetry of the superconducting order parameter.

## Main

The spin-1/2 electrons in superconductive materials can bind into a spin-zero singlet or spin-one triplet^1,2^ eigenstate. In the former case, the momentum **k** dependence of electron pairing potentials Δ(**k**) has even parity, Δ(**k**) = Δ(−**k**), whereas in the latter its parity is odd, Δ(−**k**) = −Δ(**k**). Superfluid ^3^He (ref. ^3^) is the only material whose Δ(**k**) has definitely been identified as odd parity, spin-triplet. If such a superconductor exists, the electron pair potential is a matrix $${\Delta}_{{\bf{k}}}\equiv \left(\begin{array}{cc}{\Delta}_{{\bf{k}}\uparrow \uparrow} & {\Delta}_{{\bf{k}}\uparrow \downarrow}\\ {\Delta}_{{\bf{k}}\downarrow \uparrow} & {\Delta}_{{\bf{k}}\downarrow \downarrow}\end{array}\right)$$ representing pairing with all three spin-one eigenstates (↑↑,↓↓,↑↓+↓↑), or equivalently $\Delta ({\bf{k}})\equiv \Delta ({\bf{d}}\cdot {\bf{\sigma }})i{\sigma }_{2}$ in **d**-vector notation, where ${\sigma }_{i}$ are Pauli matrices. UTe_2_ is now widely surmised^4–6^ to be such an odd-parity, spin-triplet intrinsic topological superconductor (ITS). Having *D*_2__*h*_ crystal symmetry and some degree of spin-orbit coupling, UTe_2_ could, in theory, exhibit four possible odd-parity Δ(**k**) symmetries: *A*_*u*_, *B*_1__*u*_, *B*_2__*u*_ and *B*_3__*u*_ (refs. ^6–8^). If extant, the *A*_*u*_ phase would be fully gapped and preserve time-reversal symmetry (akin to the *B* phase of superfluid ^3^He (ref. ^3^)), whereas the *B*_1__*u*_, *B*_2__*u*_ and *B*_3__*u*_ phases also preserve time-reversal symmetry and would have point nodes in Δ(**k**) along the three orthogonal lattice axes, provided a Fermi surface (FS) exists in these directions (akin to the hypothetical planar phase of superfluid ^3^He (ref. ^9^). Linear combinations of these four states might, when accidentally degenerate, break time-reversal symmetry, generating distinct chiral Δ(**k**). For UTe_2_, the challenge is to determine definitely which, if any, of these states exist.

**Fig. 1:**
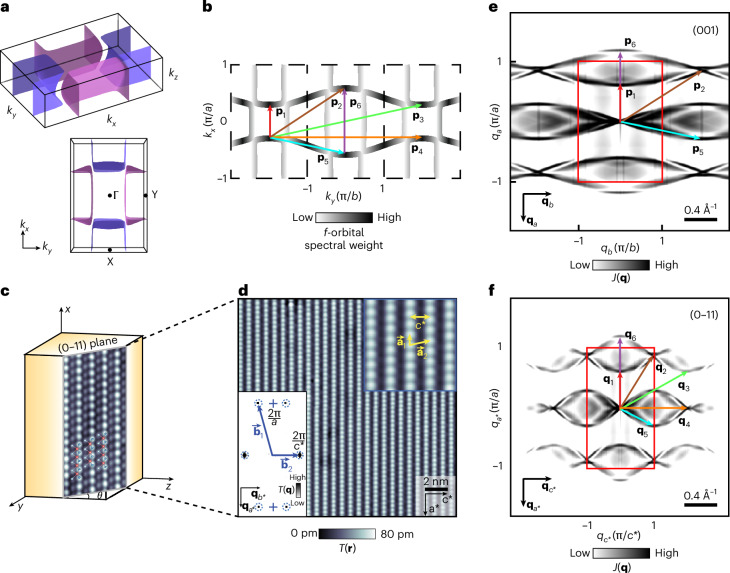
FS and QPI predictions for the (0–11) surface of UTe_2_. **a**, Bulk FS of UTe_2_ based on recent band-structure models (Methods). **b**, Bulk UTe_2_ FS intersecting the ${k}_{z}=0$ plane. Highlighted with coloured arrows are a sextet of scattering interference wavevectors **p**_*i*_*, i* = 1–6 connecting spectral weight maxima in **k** space derived heuristically from *f-*electron orbital contributions. **c**, Schematic of UTe_2_ (0–11) cleave surface, whose normal is oriented to the crystal **b**-axis at $\theta \cong 24^\circ$. Uranium (red) and two inequivalent tellurium atom sites (dark and light blue) overlaid on a *T*(**r**) image, revealing the tellurium chains of the (0–11) cleave surface. **d**, Typical topographic image *T*(**r**) of the (0–11) cleave surface of UTe_2_. Top-right inset shows both the *x*-axis unit cell distance *a* and the *y:z*-axis lattice periodicity c*, as well as the (0–11) termination surface primitive lattice vectors, **a**_1_ and **a**_2_. Bottom-left inset, *T*(**q**), Fourier transform of *T*(**r**), shows the (0–11) reciprocal unit cell. **e**, JDOS ($J({\bf{q}},E)$) calculated using the model featured in **b** for ${k}_{z}=0$ of the crystal termination layer (001). The sextet of scattering interference wavevectors **p**_*i*_*, i* = 1–6 connecting maxima in **b** are overlaid. **f**, *J*(**q***, E*) predicted for the (0–11) termination from the FS model of **b**. Rotation to the (0–11) plane corresponds to a change in *y*-axis coordinates ${{\bf{q}}}_{1,y}={{\bf{p}}}_{1,y}\sin \theta$. Here the sextet of QPI wavevectors **q**_i_*, i* = 1–6, now viewed along the normal to (0–11), are overlaid. Source data

Of course, it is the normal-state electronic structure of UTe_2_ that forms the basis upon which Δ(**k**) phenomenology emerges at lower temperatures. Atomic-resolution differential tunnelling conductance $g({\bf{r}},V\;)\equiv {{\rm{d}}I}/{{\rm{d}}V}\left({\bf{r}},V\;\right)$ imaging visualizing the density of states $N\left({\bf{r}},E\;\right)$ and its Fourier transform $g\left({\bf{q}},E\;\right)\propto N\left({\bf{q}},E\;\right)$ can be used to establish those electronic-structure characteristics. A conventional model of the bulk first Brillouin zone (BZ) of UTe_2_ sustaining a two-band FS as now widely hypothesized^6–8,10,11^ is shown in Fig. 1a, and its contours at ${k}_{z}=0$ are presented in Fig. 1b. Quantitative predictions for the normal-state quasiparticle interference (QPI) in UTe_2_ then require a Hamiltonian $${H}_{{\rm{UT}}{{\rm{e}}}_{2}}=\left(\begin{array}{cc}{H}_{\rm{U-U}} & {H}_{\rm{U-{Te}}}\\ {H}_{\rm{U-{Te}}}^{+} & {H}_{{\rm{Te}-{Te}}}\end{array}\right)$$ such that ${H}_{\rm{U}-\rm{U}}$ and ${H}_{{\rm{Te}-{Te}}}$ describe, respectively, the two uranium and tellurium orbitals and ${H}_{\rm{U}-\rm{{Te}}}$ their hybridization (Methods). In Fig. 1b, the intensity of each curve qualitatively represents the hybridized U 5*f* orbital spectral weight in the ${k}_{{\rm{z}}}$ = 0 plane determined by quantum oscillations^12^. From this, one might anticipate strong scattering interference with a sextet of wavevectors **p**_*i*_: *i* = 1–6, as indicated by the arrows (Table 1).

**Table 1:** Anticipated sextet of QPI wavevectors viewed in the (001) plane based on our band-structure model

Wavevector	p_1_	p_2_	p_3_	p_4_	p_5_	p_6_
Coordinate $\left(\frac{2\pi }{a},\frac{2\pi }{b}\right)$	(0.29, 0)	(0.43, 1)	(0.29, 2)	(0, 2)	(−0.14, 1)	(0.57, 0)

**Extended Data Fig. 1:**
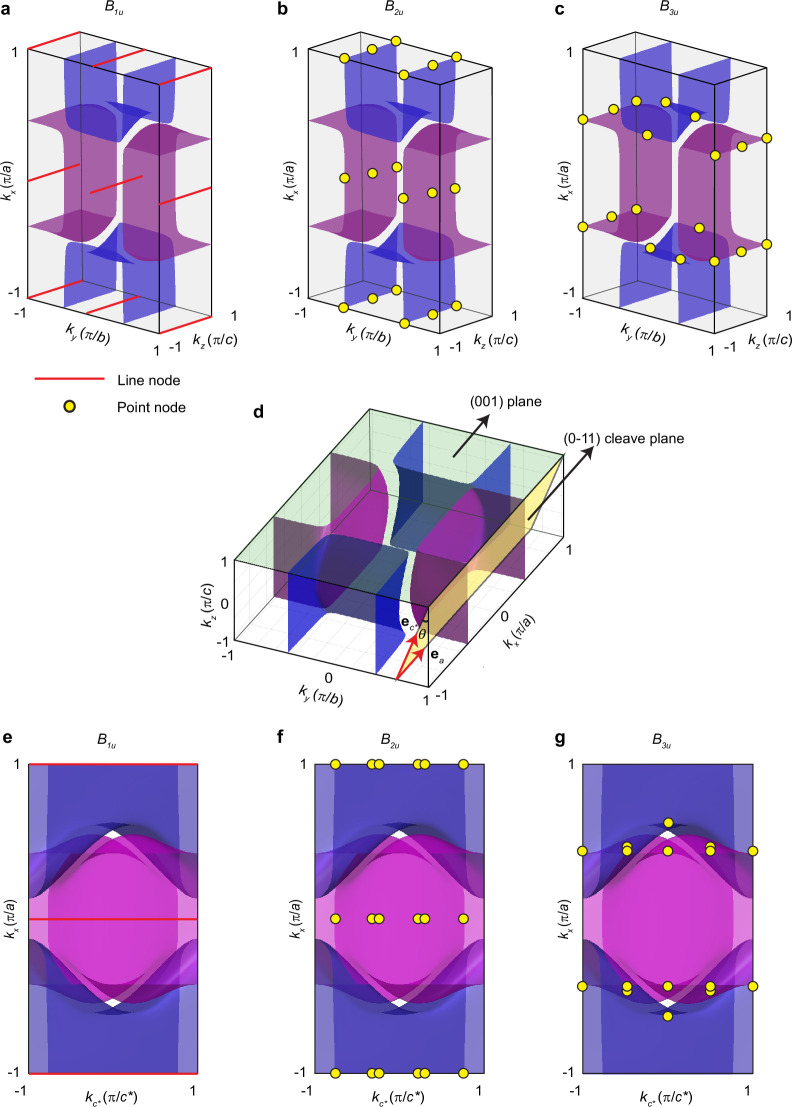
Projection of Fermi surfaces and gap nodes in *B*_*1u*_, *B*_*2u*_, and *B*_*3u*_. **a-c**. Bulk FS of UTe_2_ in 3D Brillion zone, showing the nodeless *B*_*1u*_ gap, eight independent *B*_*2u*_ gap nodes, and eight independent *B*_*3u*_ gap nodes. The red lines indicate the location where the order parameter vanishes. **d**. Projection of the (001) plane (green) onto the (0-11) plane SBZ (yellow). **e-g**. Bulk FS of UTe_2_ projection onto the (0-11) plane SBZ, showing the *B*_*1u*_ gap nodal lines, *B*_*2u*_ gap nodes, and *B*_*3u*_ gap nodes. Source data

However, the natural cleave surface of UTe_2_ crystals is not (001) but rather^13^ (0–11), here shown schematically in Fig. 1c. It is this surface that the scan-tip approaches perpendicularly. Its lattice vectors ${{\bf{a}}}_{1}$, ${{\bf{a}}}_{2}$ are identified in the top-right inset of Fig. 1d alongside the intertellurium chain distance ${c}^{{\boldsymbol{* }}}=0.76$ nm. The corresponding reciprocal lattice vectors ${{\bf{b}}}_{1}$, ${{\bf{b}}}_{2}$ are shown in the bottom-left inset of Fig. 1d, which is $T\left({\bf{q}}\right),$ the Fourier transform of $T({\bf{r}})$. To clarify the normal-state band structure and quasiparticle interference viewed from the (0–11) plane, in Fig. 1e we first present the **k**-space joint density of states (JDOS) $J({\bf{q}},E)$ calculated at the (001) plane using our UTe_2_ FS that takes into account the uranium *f-*orbital spectral weight (Fig. 1b). The sextet of scattering wavevectors **p**_*i*_: *i* = 1–6 derived heuristically above are revealed as primary peaks in $J({\bf{q}},E)$. In Fig. 1f, we present $J({\bf{q}},E)$ for the same band-structure model but viewed along the normal to the (0–11) plane (Methods and Extended Data Fig. 1). Here the *y* coordinates of the (0–11) sextet become ${{\bf{q}}}_{1,y}={{\bf{p}}}_{1,y}\sin \theta$, where $\theta =$24°and ${c}^{* }$ is the (0–11) surface *y*:*z*-axis lattice periodicity (coloured arrows in Fig. 1f) (Table 2).

**Table 2:** Anticipated sextet of QPI wavevectors viewed in the (0–11) plane based on our band-structure model

Wavevector	q_1_	q_2_	q_3_	q_4_	q_5_	q_6_
Coordinate $\left(\frac{2\pi }{a},\frac{2\pi }{{c}^{* }}\right)$	(0.29, 0)	(0.43, 0.5)	(0.29, 1)	(0, 1)	(−0.14, 0.5)	(0.57, 0)

**Extended Data Fig. 2:**
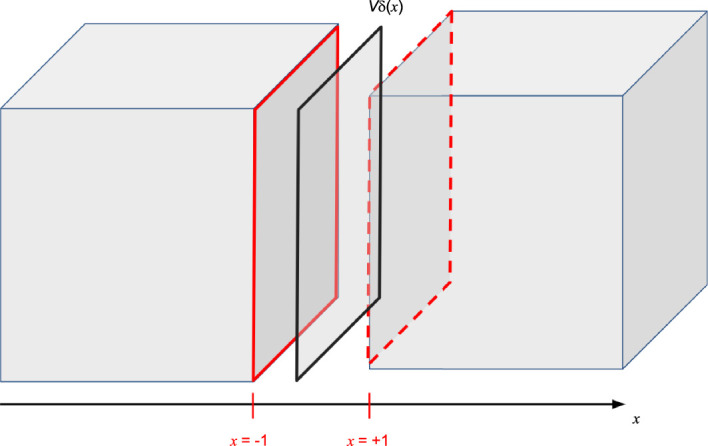
Schematics of the 3D system and the technique to compute the surface Green’s functions. The black parallelogram denotes the planar-impurity-potential which is oriented parallel to the (0-11) crystal plane for all calculations, while the red ones correspond to the two created surfaces on the neighboring planes at $x=\pm 1$, one lattice constant away from the impurity plane.

**Extended Data Fig. 3:**
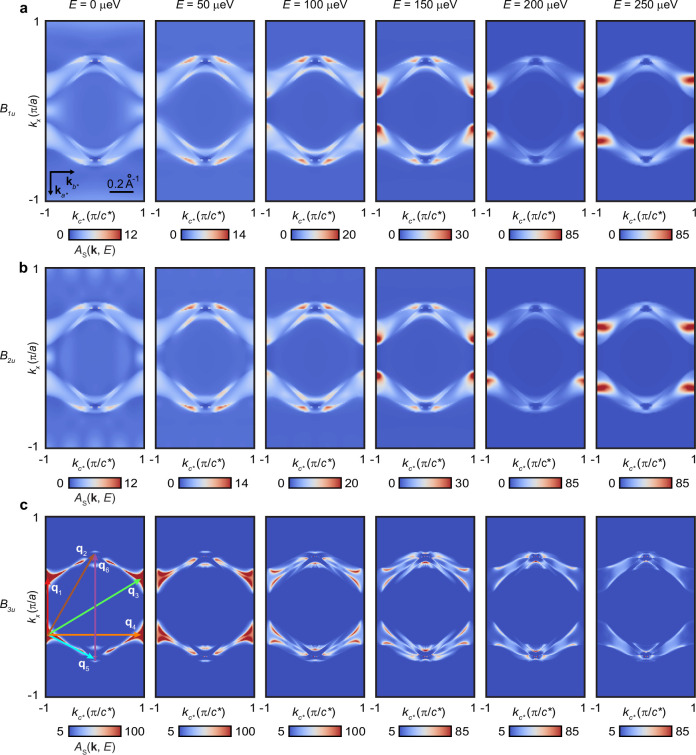
Surface spectral function for *B*_*1u*_, *B*_*2u*_ and *B*_*3u*_. **a**. Surface spectral function *A*_s_(**k**, *E*) for *B*_*1u*_ at the (0-11) SBZ. **b**. Surface spectral function *A*_s_(**k**, *E*) for *B*_*2u*_ at the (0-11) SBZ. **c**. Surface spectral function *A*_s_(**k**, *E*) for *B*_*3u*_ at the (0-11) SBZ. The anticipated sextet of scattering wavevectors **q**_*i*_, *i* = 1-6 are overlaid. Source data

**Extended Data Fig. 4:**
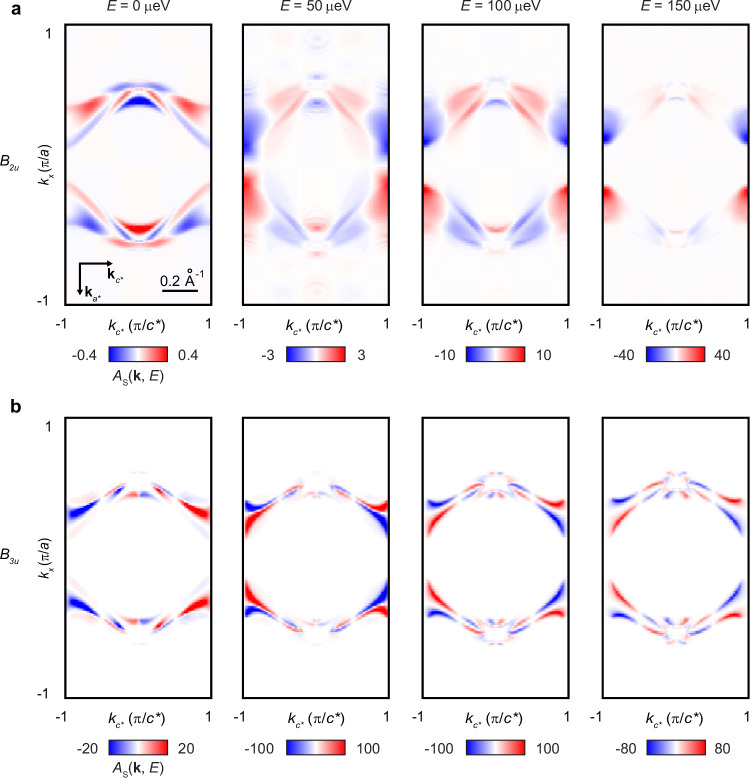
Spin-resolved surface spectral function for *B*_*2u*_ and *B*_*3u*_ gap symmetry. **a**. Spin-resolved surface spectral function at the (0-11) SBZ of UTe_2_ for a *B*_*2u*_ gap function. Spin oriented parallel to the crystal **b**-axis are denoted by red and antiparallel by blue. **b**. Spin-resolved surface spectral function for a *B*_*3u*_ gap function. Spin parallel to the crystal **a**-direction is denoted by red and spin antiparallel to the **a**-direction is denoted by blue. ${{\bf{q}}}_{1}$ scattering is distinct for *B*_*3u*_ gap symmetry, but is not favored for *B*_*2u*_ gap symmetry because spin-flip scattering processes are forbidden, as illustrated in main text Fig. 4 g, i. Source data

**Extended Data Fig. 5:**
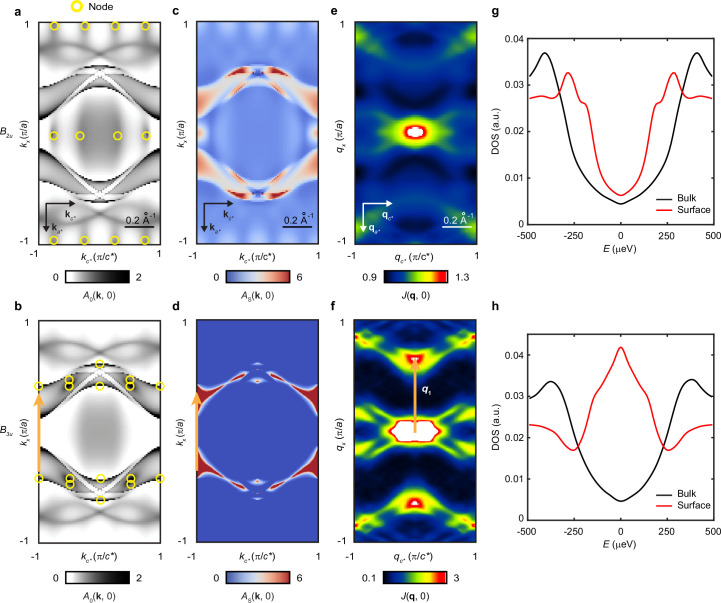
JDOS, QPI and DOS simulations for *B*_*2u*_ and *B*_*3u*_ gap symmetry. **a-b**. Bulk spectral function of UTe_2_ projection at the (0-11) SBZ of UTe_2_ for *B*_*2u*_ and *B*_*3u*_ gap structures. **c-d**. Surface spectral function *A*_s_(**k**, 0) at the (0-11) SBZ of UTe_2_. **e-f**. Simulated QPI using the *J*(**q**, 0) at the (0-11) SBZ of UTe_2_. ${{\bf{q}}}_{1}$ scattering is distinct for *B*_*3u*_ gap symmetry. **g-h**. Bulk and surface band DOS calculations for (**g**) *B*_*2u*_ and (**h**) *B*_*3u*_ with energy gap $\Delta =300\mu {\rm{eV}}$. Both gap symmetries show similar bulk DOS. At the (0-11) surface, the *B*_*3u*_ surface state contributes greatly to the zero-energy DOS while the *B*_*2u*_ surface state is expected to have a much weaker contribution. Source data

This QPI sextet ${{\bf{q}}}_{i}$ is quantitatively consistent with the precise $N({\bf{q}},E)$ and $J({\bf{q}},E)$ calculations carried out using the procedures outlined in Methods and Extended Data Figs. 2–4 and the results presented in Extended Data Fig. 5 and is pivotal to the remainder of our study.

Classically, odd-parity superconductors should exhibit zero-energy surface Andreev bound states^14–18^ (SABS), which are generated by the universal π phase shift during Andreev reflections from the odd-parity ${\Delta }_{{\bf{k}}}$ (Methods). Hence, observation of a zero-energy SABS at an arbitrary crystal surface of a superconducting material would indicate that its ${\Delta }_{{\bf{k}}}$ has odd parity. More intriguingly, intrinsic bulk topological superconductivity^19,20^ exists most simply in the case of odd-parity spin-triplet superconductors. A definitive characteristic^21,22^ of such an ITS would be a topological quasiparticle surface band (QSB) with momentum-energy relationship **k**(*E*) existing only for energies ${|E|}\le \Delta$ within the maximum superconducting energy gap^21,23–34^. In UTe_2_, there is now firm evidence from the pronounced zero-energy Andreev conductance^35^ for the presence of a QSB at the (0–11) surface. Hence, QPI visualization studies and analyses for UTe_2_ must take cognizance of the **k**-space structure of any such QSB.

**Fig. 2:**
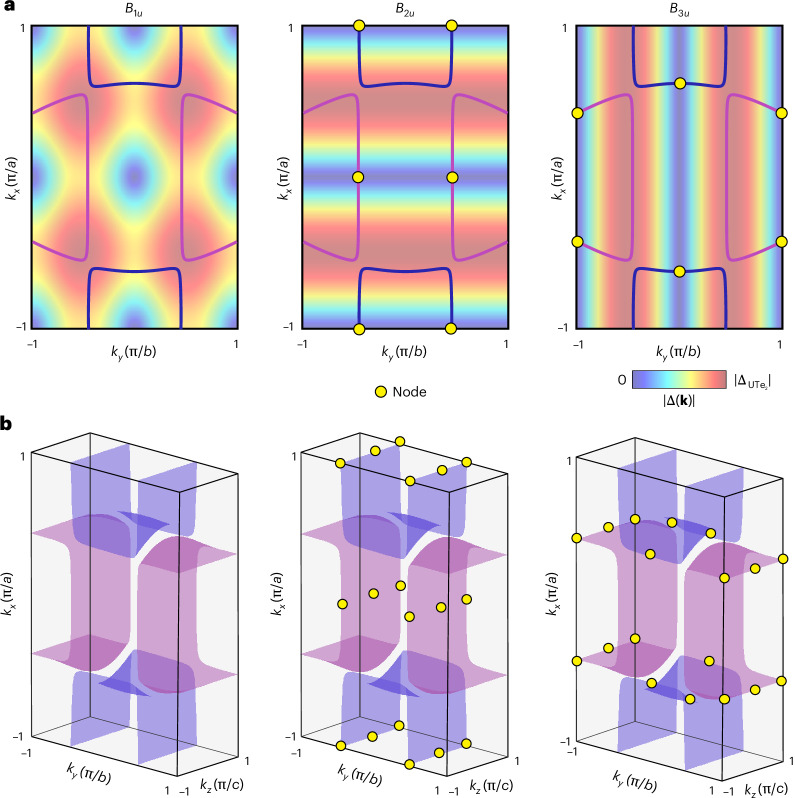
Simple models for UTe_2_ Δ_**k**_. **a**, Magnitude of the UTe_2_ superconductive energy gap $|{\Delta }_{{\bf{k}}}|$ at ${k}_{z}=0$ for the *B*_1__*u*_*, B*_2__*u*_ and *B*_3__*u*_ order parameters on the FS shown in Fig. 1a,b. The nodal locations occur within the dark blue regions where $|{\Delta }_{{\bf{k}}}|\to 0$. Note that *B*_1__*u*_ does not exhibit gap nodes in this model because the FS is open along the *k*_*z*_ axis. **b**, From **a**, the theoretically predicted nodal locations for the *B*_1__*u*_*, B*_2__*u*_ and *B*_3__*u*_ order parameters on the FS shown in Fig. 1a,b are indicated by yellow dots. For *B*_1__*u*_, ${\bf{d}}\propto (\sin {k}_{y}b,\sin {k}_{x}a,0)$ and zeros occur at ${k}_{y}=0,\pm \frac{\pi }{b}$, ${k}_{x}=0,\pm \frac{\pi }{a}$; for *B*_2__*u*_, ${\bf{d}}\propto (\sin {k}_{z}c,0,\sin {k}_{x}a)$ and zeros occur at ${k}_{z}=0,\pm \frac{\pi }{c},{k}_{x}=0,\pm \frac{\pi }{a}$; and for *B*_3__*u*_, ${\bf{d}}\propto (0,\sin {k}_{z}c,\sin {\rm{}}{k}_{y}b)$ and zeros occur at ${k}_{z}=0,\pm \frac{\pi }{c}$, ${k}_{y}=0,\pm \frac{\pi }{b}$ (Methods and Extended Data Fig. 1). Alternative gap functions and consequent nodal locations are discussed in Methods, Table 3 and Extended Data Fig. 7. Source data

In that context, we next consider Bogoliubov QPI imaging, a recognized technique for Δ(**k**) determination in complex superconductors^21,36–43^, in the superconducting state at temperatures much lower than the UTe_2_ superconducting transition temperature. In this material, the *A*_*u*_ state should be completely gapped on both FSs, whereas *B*_1__*u*_, *B*_2__*u*_ and *B*_3__*u*_ states could exhibit point nodes along the *k*_*z*_ axis, *k*_*y*_ axis and *k*_*x*_ axis, respectively. These bulk in-gap Bogoliubov eigenstates are described by the dispersion

1 $${E}_{{\bf{k}}}=\sqrt{{\xi }_{{\bf{k}}}^{2}+{\Delta }^{2}({|{\bf{d}}\left({\bf{k}}\right)|}^{2}\pm |{\bf{d}}({\bf{k}})\times {{\bf{d}}}^{* }({\bf{k}})|)}$$

so that **k**-space locations of energy-gap zeros are defined in general by $|{\bf{d}}\left({\bf{k}}\right){|}^{2}\pm |{\bf{d}}\left({\bf{k}}\right)\times {{\bf{d}}}^{* }\left({\bf{k}}\right)|=0$. Formally, *A*_*u*_ is fully gapped (nodeless). Modelling the pair potential magnitude |Δ_**k**_| for each order parameter throughout the ${k}_{z}=0$ (001) BZ in Fig. 2a yields nodes at the dark blue regions where |Δ(**k**)| approaches 0. Thus, although *A*_*u*_ supports no energy-gap nodes by definition and *B*_1__*u*_ exhibits no energy-gap nodes in this model, there are numerous nodes in highly distinct **k**-space locations for *B*_2__*u*_ and *B*_3__*u*_. Figure 2b presents a schematic of the bulk FS with energy-gap nodal locations for *B*_1__*u*_, *B*_2__*u*_ and *B*_3__*u*_ from equation (3) shown as yellow dots.

**Extended Data Fig. 6:**
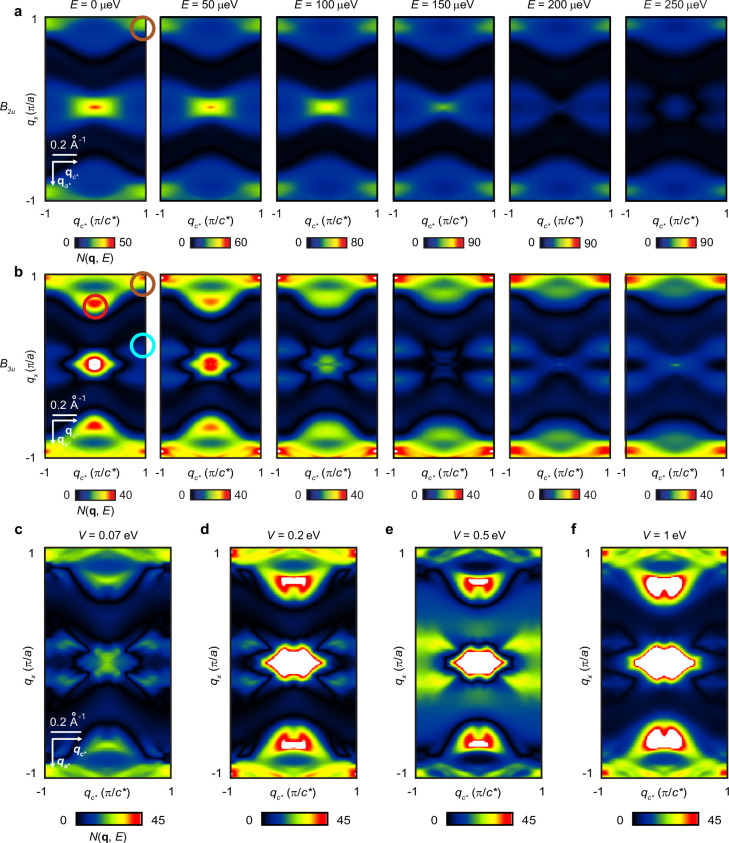
QPI simulations with 2D Gaussian background caused by the ‘aperture’ of the scan tip, and QPI simulations for *B*_*3u*_ order parameter using different scattering potentials. **a**. Unfiltered QPI simulations $N({\bf{q}},E)$ for a *B*_*2u*_-QSB at the (0-11) SBZ of UTe_2_. The existing QPI wavevector ${{\bf{q}}}_{2}$ is identified as the maxima position (brown circle). **b**. Unfiltered QPI simulations $N({\bf{q}},E)$ for a *B*_*3u*_-QSB at the (0-11) SBZ of UTe_2_. Each existing QPI wavevector, ${{\bf{q}}}_{1}$ (red), ${{\bf{q}}}_{2}$ (brown) and ${{\bf{q}}}_{5}$ (cyan), is identified as the maxima position (colored circles). To generate Fig. 4 g, i of the main text, these images are multiplied by using a 2D circular Gaussian function. **c-f**. QPI simulations are performed using scattering potentials of *V* = 0.07 eV, 0.2 eV, 0.5 eV, and 1 eV. Across this range, the predicted scattering wavevectors remain highly consistent. Varying the scattering potentials only leads to the change of the relative amplitudes of the wavevectors. The typical $N({\bf{q}},50{\rm{\mu }}{\rm{eV}})$ are presented here and $\eta =30{\rm{\mu }}{\rm{eV}}$ are used in the calculations. Source data

Under these circumstances, to generate QPI predictions for the QSB in UTe_2_, we use the Hamiltonian

2 $$H\left({\bf{k}}\right)=\left(\begin{array}{cc}{H}_{{\rm{UT}}{{\rm{e}}}_{2}}\left({\bf{k}}\right)\bigotimes {I}_{2} & \Delta \left({\bf{k}}\right)\bigotimes {I}_{4}\\ {\Delta }^{+}\left({\bf{k}}\right)\bigotimes {I}_{4} & -{H}_{{\rm{UT}}{{\rm{e}}}_{2}}^{* }\left(-{\bf{k}}\right)\bigotimes {I}_{2}\end{array}\right)$$

where the order parameter is $\Delta \left({\bf{k}}\right)={\Delta }_{0}\left({\bf{d}}\cdot {\bf{\upsigma }}\right)i{\sigma }_{2}$ and ${I}_{2},$ ${I}_{4}$ are the unit matrices. We consider the order parameters ${A}_{u}$, ${B}_{1u}$, ${B}_{2u}$ and ${B}_{3u}$ (Methods, Table 3 and Extended Data Fig. 1), but because ${A}_{u}$ and ${B}_{1u}$ are non-nodal, here we focus primarily on ${B}_{2u}$ and ${B}_{3u}$:

3a $${{\bf{d}}}_{{B}_{2u}}=\left({C}_{1}\sin \left({k}_{z}c\right),\,{C}_{0}\sin \left({k}_{x}a\right)\sin \left({k}_{y}b\right)\sin \left({k}_{z}c\right),{C}_{3}\sin \left({k}_{x}a\right)\right)$$

3b $${{\bf{d}}}_{{B}_{3u}}=\left({C}_{0}\sin \left({k}_{x}a\right)\sin \left({k}_{y}b\right)\sin \left({k}_{z}c\right),{C}_{2}\sin \left({k}_{z}c\right),{C}_{3}\sin \left({k}_{y}b\right)\right)$$

where *a*, *b*, *c* are lattice constants, and ${C}_{0}=0,{C}_{1}=300$ μeV, ${C}_{2}=300$ μeV and ${C}_{3}=300$ μeV. The unperturbed bulk Green’s function is then *G*_0_(**k**, *E*) = [(*E* + *iη*)*I* −*H*(**k**)]^−1^ (*η* = 100 μeV) with the corresponding unperturbed spectral function *A*_0_(**k**, *E*) = −1/π Im *G*_0_(**k**, *E*). Although obtaining the *G*_0_(**k**, *E*) is straightforward, calculating the (0–11) surface Green’s functions ${G}_{{\rm{s}}}\left({\bf{k}},{E}\right)$ and spectral functions *A*_S_(**k**, *E*) is substantially more difficult. The surface Green’s function characterizes a semi-infinite system with broken translation symmetry and therefore cannot be calculated directly. Here we use a technique in which we model the surface using a strong planar impurity^44–46^. In the limit of an infinite impurity potential, the impurity plane splits the system into two semi-infinite spaces. Then only wavevectors in the (0–11) plane remain good quantum numbers. The effect of the planar impurity can then be exactly calculated using the T-matrix formalism, which gives one access to the surface Green’s function of the semi-infinite system. Details of this procedure can be found in Methods and Extended Data Fig. 2. The predicted surface quasiparticle spectral function, ${A}_{{\rm{S}}}\left({\bf{k}},E\right)$, calculated using the above method for the *B*_1__*u*_, *B*_2__*u*_ and *B*_3__*u*_ order parameters, also appears in Methods and Extended Data Fig. 3. For Bogoliubov QPI predictions at the (0–11) surface of UTe_2_, we use a localized impurity potential $\hat{V}=V{\tau }_{z}\otimes {I}_{8}$, where *V* = 0.2 eV (Methods and Extended Data Fig. 6) and determine the exact solution for the perturbed generalized surface Green’s function ${g}_{{\rm{S}}}\left({\bf{q}},{\bf{k}},E\right)$ using the T-matrix $T\left(E\right)={(I-\hat{V}\int \frac{{d}^{2}{\bf{k}}}{{S}_{{BZ}}}{G}_{s}\left({\bf{k}},E\right))}^{-1}\hat{V}$. Then the QPI patterns for the UTe_2_ QSB are predicted directly using

4 $$N\left({\bf{q}},E\right)=\frac{i}{2\pi }\int \frac{{d}^{2}{\boldsymbol{k}}}{{S}_{{BZ}}}{Tr}\left[{g}_{S}({\bf{q}},{\bf{k}},E)\right]$$

where

5 $${g}_{{\rm{S}}}\left({\bf{q}},{\bf{k}},E\right)={G}_{s}\left({\bf{q}},E\right)T\left(E\right){G}_{s}\left({\bf{q}}-{\bf{k}},E\right)-{G}_{s}^{* }\left({\bf{q}}-{\bf{k}},E\right){T}^{* }\left(E\right){G}_{s}^{* }({\bf{q}},E)$$

**Extended Data Fig. 7:**
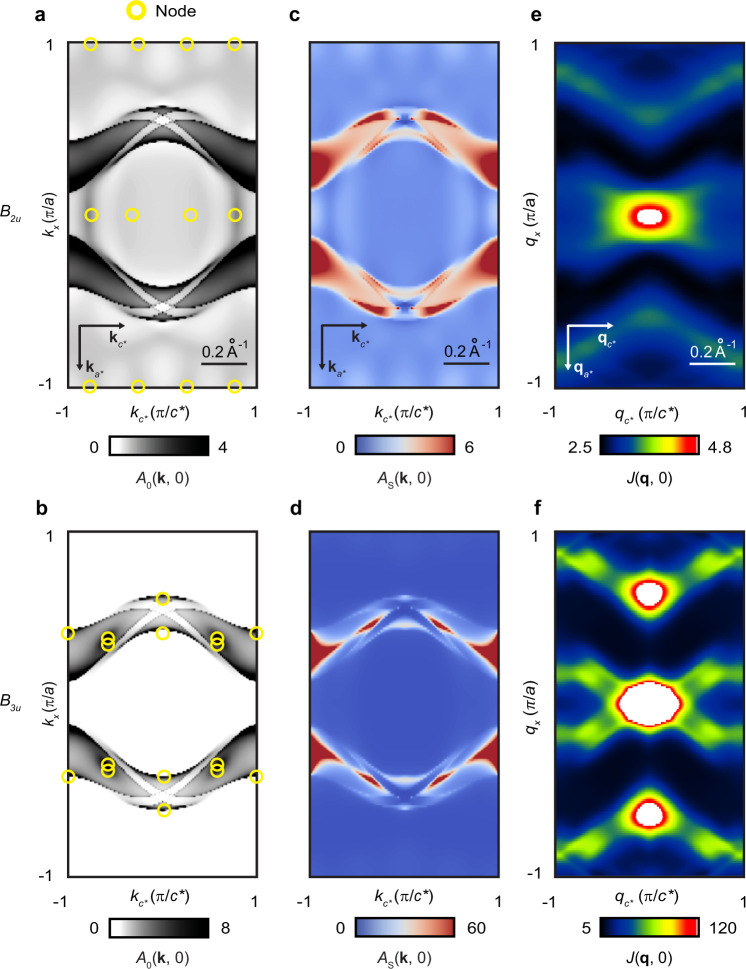
QPI simulations for *B*_*2u*_ and *B*_*3u*_ gap symmetry with alternative d-vector. **a**. Bulk projected spectral function, ${A}_{0}({\bf{k}},0)$ of the Fermi surface model described above with an alternative, symmetry-allowed *B*_*2u*_ **d**-vector. The locations of projected nodes are highlighted with yellow circles. **b**. ${A}_{0}({\bf{k}},0)$ at the (0-11) plane with an alternative **d**-vector of *B*_*3u*_ symmetry. The nodal locations in **a** and **b** are very similar to those obtained using the **d**-vectors in Methods Table 3. **c**. Surface spectral function ${A}_{{\rm{s}}}({\bf{k}},0)$ for the alternative *B*_*2u*_ **d**-vector. The QSB occupies regions connecting the projection of bulk nodes. **d**. ${A}_{s}({\bf{k}},0)$ for the alternative *B*_*3u*_ **d**-vector. The QSB develops on similar regions of the SBZ as in the Main Text. **e**. Joint density of states *J*(**q**, 0) of **c** for *B*_*2u*_. **f**. Joint density of states *J*(**q**, 0) of **d** for *B*_*3u*_. Note ${{\bf{q}}}_{1}$ scattering remains distinct for *B*_*3u*_ gap symmetry. Source data

By calculating the trace over particle-hole space on ${g}_{{\rm{S}}}\left({\bf{q}},{\bf{k}},E\right)$, the obtained $N\left({\bf{q}},E\right)$ is in general a complex quantity; all simulations presented herein are therefore ${|N}({\bf{q}},E)|$. The predicted QSB spectral function, ${A}_{{\rm{S}}}\left({\bf{k}},E\right)$, JDOS $J({\bf{q}},0)$ and density of states spectra for a *B*_2__*u*_ QSB and *B*_3__*u*_ QSB within the (0–11) SBZ appear in Methods and Extended Data Fig. 5. We further take into account the **q**-space sensitivity of our scan tip by applying a two-dimensional (2D) Gaussian filter to the $N\left({\bf{q}},E\right)$ calculated using equation (4) (Methods and Extended Data Fig. 6). Additionally, we discuss alternative, symmetry-allowed, gap structure models and derive their resulting ${A}_{0}\left({\bf{k}},E\right)$, ${A}_{{\rm{S}}}\left({\bf{k}},E\right)$ and $J({\bf{q}},0)$ (Methods, Table 4 and Extended Data Fig. 7), finding them indistinguishable from the results presented in Extended Data Fig. 5. Ultimately, the existence of these specific QPI characteristics in UTe_2_ would provide strong confirmation of both a superconductive QSB and its foundational odd-parity bulk order parameter.

**Fig. 3:**
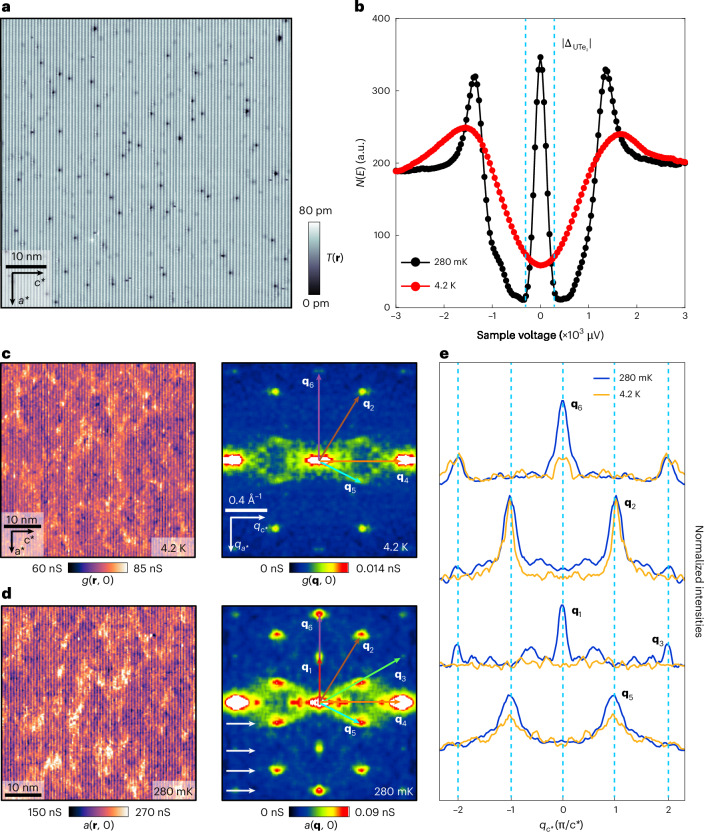
QPI visualization of UTe_2_ superconductive QSB. **a**, Typical topographic image *T*(**r**) of the (0–11) cleave surface of UTe_2_. **b**, Measured differential conductance in the UTe_2_ normal-state *g*(*V*) at *T* = 4.2 K; and Andreev differential conductance in the superconducting state *a*(*V*) at *T* = 280 mK. Intense Andreev conductance is observed at *V* = 0. **c**, Measured *g*(**r**, 0) and *g*(**q**, 0) at *T* = 4.2 K in the UTe_2_ normal state in the identical FOV as in **a**. The setpoint is *V*_s_ = 3 mV and *I* = 200 pA. **d**, Measured *a*(**r**, 0) and *a*(**q**, 0) at *T* = 280 mK in the UTe_2_ superconducting state in the identical FOV as in **a** and **c**. Here a sextet of scattering interference wavevectors **q**_*i*_, *i* = 1–6 from theoretical predictions are overlaid. The excellent correspondence between the predictions and the measured QPI data is striking, with all theory and experiment wavevectors **q**_1_, **q**_2_, **q**_3_, **q**_4_, **q**_5_ and **q**_6_ being within 3% of each other. This experimental detection of the sextet has been repeated multiple times (Methods and Extended Data Fig. 10). The set point is *V*_s_ = 3 mV and *I* = 200 pA. **e**, Relative amplitudes of the sextet wavevectors in the normal and superconducting states. Comparison of *g*(**q**, 0) linecuts at *T* = 4.2 K and *a*(**q**, 0) linecuts measured *T* = 280 mK. The linecuts are taken horizontally in the **q** space indicated by white arrow in **d**. The linecuts have been normalized by their background intensities at 280 mK and 4.2 K. The intensities of **q**_5_ and **q**_6_ have been greatly enhanced in the superconducting state. Most importantly, **q**_1_ only appears in the superconducting state. Source data

**Extended Data Fig. 8:**
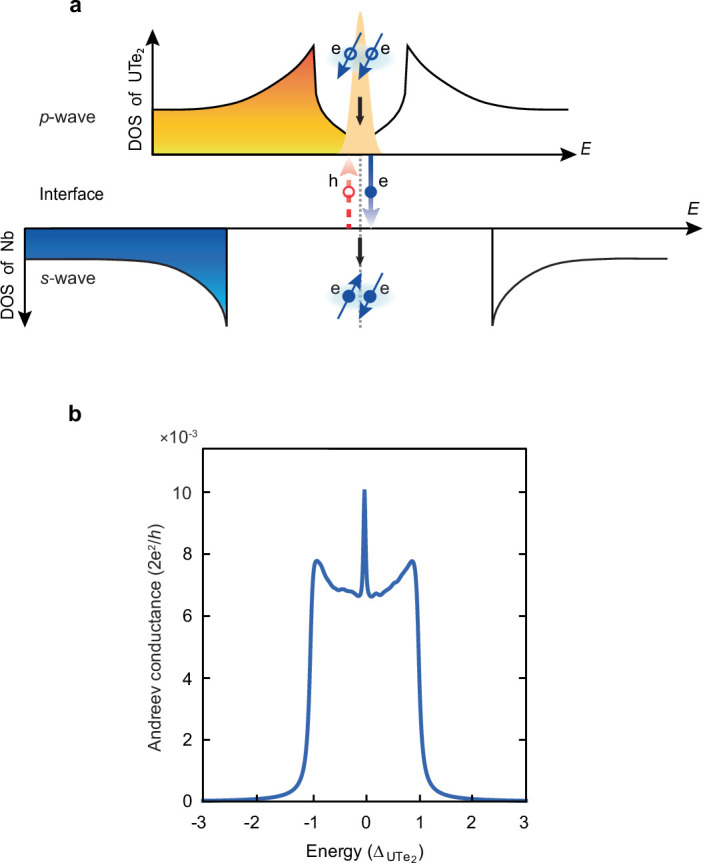
QSB generated Andreev conductance within SIP model. **a**. Schematic of the UTe_2_ QSB and Andreev tunneling to the *s*-wave electrode, through a two quasiparticle transport process. **b**. Calculated Andreev conductance *a*(*V*) in the SIP model. The SIP model predicts a sharp peak in Andreev conductance surrounding zero-bias if the QSB is that of a *p*-wave, nodal, odd-parity superconductor that mediates the *s*-wave to *p*-wave electronic transport processes. Source data

**Extended Data Fig. 9:**
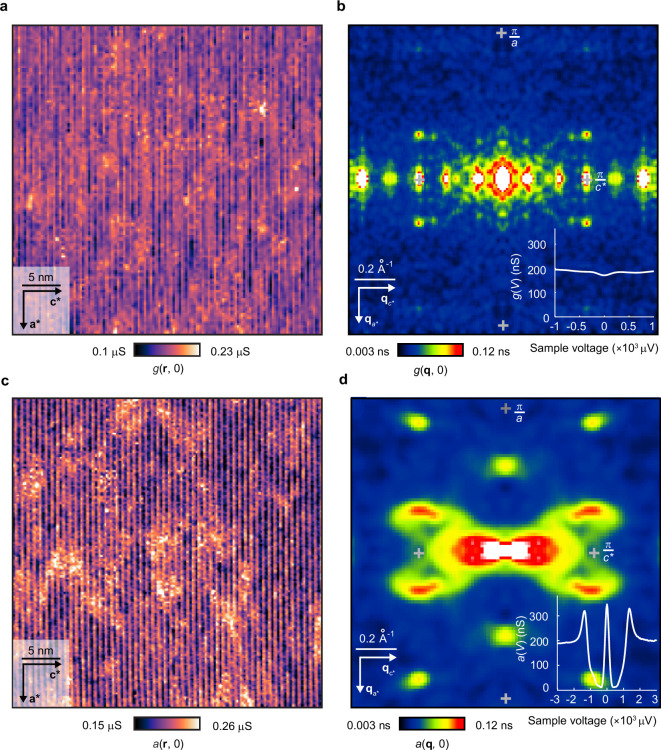
Non-superconductive tip and superconducting tip QSB scattering interference detection. **a**. Measured normal-tip *g*(**r**, 0) at *T* = 280 mK. **b**. Measured normal tip *g*(**q**, 0) at *T* = 280 mK. Inset: Normal-tip single-electron tunneling spectrum *g*(*V*). **c**. Measured superconductive-tip *a*(**r**, 0) at *T* = 280 mK. **d**. Measured superconductive-tip *a*(**q**, 0) at *T* = 280 mK. Inset: superconductive-tip Andreev tunneling spectrum *a*(*V*) as described in detail in ref. ^35^. Source data

Experimental exploration of such phenomena is challenging in UTe_2_, and several key technical advances were employed to improve on previous studies^35^. First we identified regions where the QPI signal predominantly originates from a single type of identical impurity (Te_2_ vacancies); second, the field of view (FOV) studied here is larger thus improving the **q**-space resolution; third, by using Andreev tunnelling, the energy resolution is ~10 μeV (ref. ^13^) and the QPI signal-to-noise ratio is strongly enhanced (see below). Figure 3a then shows a typical 66 nm square FOV topography of the (0–11) cleave surface, which can be studied in both the normal and superconducting states. Figure 3b shows characteristic d*I*/d*V* spectra measured with a superconductive tip in both the normal state at 4.2 K and the superconducting state at 280 mK, far below *T*_C_. In the latter case, two intense joint-coherence peaks are located at $$E={\Delta }_{{\rm{Nb}}}+{\Delta }_{\text{UT}{\text{e}}_{2}}$$. More importantly, a high density of QSB quasiparticles allows efficient creation/annihilation of Cooper pairs in both superconductors, thus generating intense Andreev differential conductance^35^ $$a({\bf{r}},V)\equiv {{\rm{d}}I}/{{\rm{d}}V}{|}_{A}\left({\bf{r}},V\right)$$ for $$\left|V\right| < {\Delta }_{\text{UT}{\text{e}}_{2}}/$$$${\rm{e}} \sim 300$$ μV, as indicated by blue vertical dashed lines (Methods and Extended Data Fig. 8). Compared to conventional normal-insulating-superconducting (NIS) tunnelling using a normal metallic tip (Methods and Extended Data Fig. 9), this Andreev conductance provides a substantial improvement in the energy resolution ($$\delta E \sim 10$$ µeV) of QSB scattering interference measurements. Comparing measured $$g\left({\bf{r}},V\right)$$:$$g\left({\bf{q}},V\right)$$ recorded in the normal state at 4.2 K (Fig. 3c) with measured $$a\left({\bf{r}},V\right):a\left({\bf{q}},V\right)$$ in the superconducting state at 280 mK (Fig. 3d), with both identical FOV and junction characteristics, allows determination of which phenomena at the (0–11) surface emerge only due to superconductivity. Some peaks of the sextet are present in the normal state $$g\left({\bf{q}},V\right)$$ in Fig. 3c as they originate from scattering of the normal-state band structure (Fig. 1b). The experimentally obtained normal-state QPI differs from the $$J(\bf{q},0)$$ calculations in Fig. 1f, as the former depends on spin and orbital selection rules, whereas the latter is dependent only on the geometry of the bulk band structure. Instead, the complete predicted QPI sextet **q**_*i*_: *i* = 1–6 are only detected in the superconducting state and appears to rely on scattering between QSB states. The sextet wavevectors are highlighted by coloured arrows in Fig. 3d. The experimental maxima in $$a\left({\bf{q}},V\right)$$ and the theoretically predicted $${{\bf{q}}}_{i}$$ from Fig. 1f are in excellent quantitative agreement with a maximum 3% difference between all their wavevectors. This demonstrates that the FS that dominates the bulk electronic structure of UTe_2_ is also what controls QSB **k**-space geometry at its cleave surface. Furthermore, Fig. 3e reveals how the amplitudes of the superconducting state QPI are enhanced compared to the normal-state measurements. The predominant effects of bulk superconductivity are the strongly enhanced arc-like scattering intensity connecting $${\bf{q}}=0$$ and $${{\bf{q}}}_{5}$$ and the unique appearance of purely superconductive QPI at wavevector $${{\bf{q}}}_{1}$$.

**Fig. 4:**
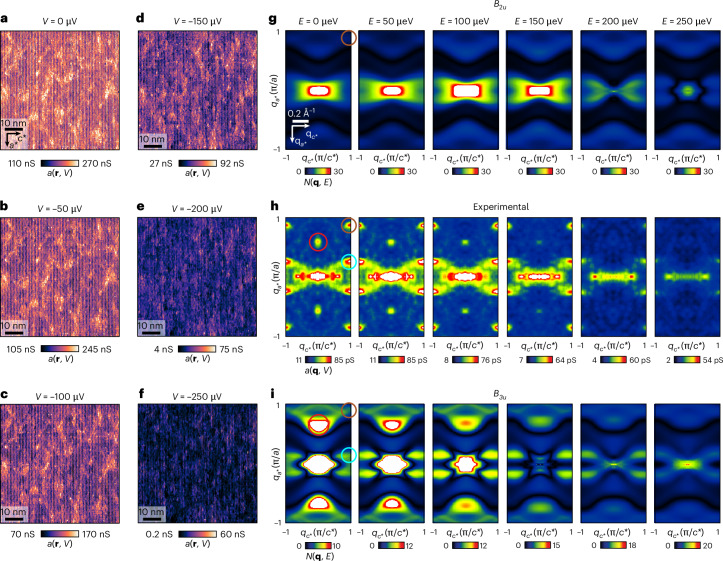
QSB QPI for Δ(**k**) identification in UTe_2_. **a**–**f**, Measured *a*(**r**, *V*) at the (0–11) cleave plane of UTe_2_ at bias voltages $\left|V\right|=$ 0 µV (**a**), 50 µV (**b**), 100 µV (**c**), 150 µV (**d**), 200 µV (**e**) and 250 µV (**f**). The setpoint is *V*_s_ = 3 mV and *I* = 200 pA. **g**, Predicted QPI patterns for a *B*_2__*u*_ QSB at the (0–11) SBZ of UTe_2_ at energies $\left|E\right|=$ 0, 50, 100, 150, 200 and 250 µeV (Methods and Extended Data Figs. 3–6). We take into account the finite radius of the scan tip in simulations by applying a 2D Gaussian to the $N\left({\bf{q}},E\right)$ maps (Methods and Extended Data Fig. 6). The existing QPI wavevector ${{\bf{q}}}_{2}$ is identified as the maxima position (brown circle) in the QPI simulation. **h**, Measured *a*(**q**, *V*) at the (0–11) cleave plane of UTe_2_ at bias voltages $\left|V\right|=$ 0, 50, 100, 150, 200 and 250 µV. The setpoint is *V*_s_ = 3 mV and *I* = 200 pA. These QPI data are derived by Fourier transformation of *a*(**r**, *V*) data in **a**–**f**. Each QPI wavevector in this FOV, ${{\bf{q}}}_{1}$ (red), ${{\bf{q}}}_{2}$ (brown) and ${{\bf{q}}}_{5}$ (cyan), is identified as the maxima position (coloured circles) in the experimental QPI data. In particular, ${{\bf{q}}}_{1}$ is a characteristic only of the *B*_3__*u*_ superconducting state, and it only exists inside the energy gap. ${{\bf{q}}}_{1}$ cannot be due to a pair density wave (Methods). **i**, Predicted QPI patterns for a *B*_3__*u*_ QSB at the (0–11) SBZ of UTe_2_ at energies $\left|E\right|=$ 0, 50, 100, 150, 200 and 250 µeV (Methods and Extended Data Figs. 3–6). Each QPI wavevector, ${{\bf{q}}}_{1}$, ${{\bf{q}}}_{2}$ and ${{\bf{q}}}_{5}$, is identified as the maxima position (coloured circles) in the QPI simulation. We take into account the finite radius of the scan tip in simulations by applying a 2D Gaussian to the $N\left({\bf{q}},E\right)$ maps (Methods and Extended Data Fig. 6). Source data

**Extended Data Fig. 10:**
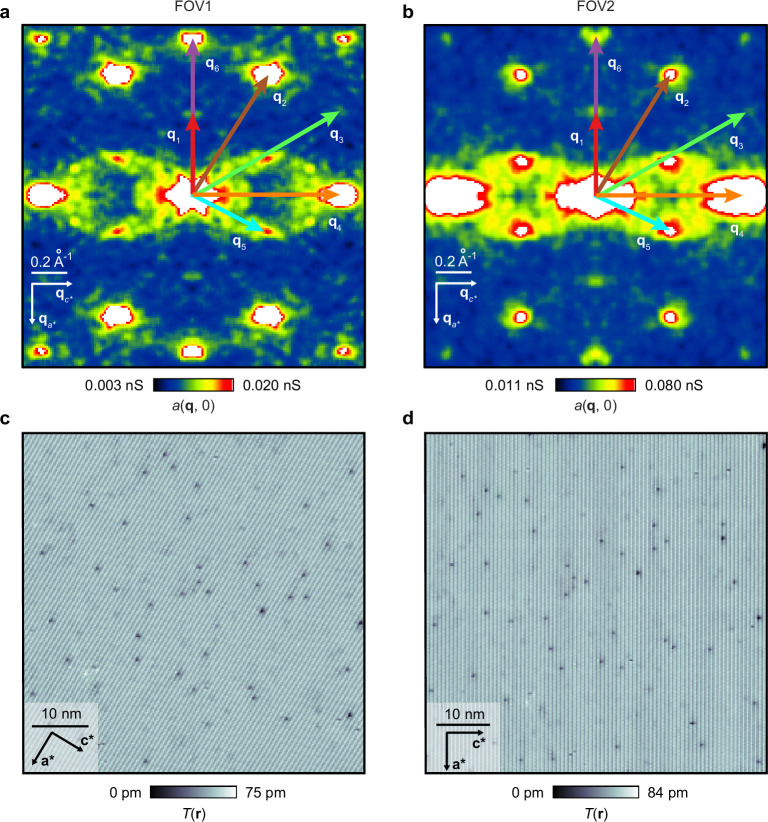
Independent QSB QPI visualization experiments. **a**-**b**. Two independent measurements of *a*(**q**, 0) at *T* = 280 mK confirm the repeatability of the sextet of scattering wavevectors for the *B*_*3u*_-QSB. **c**-**d**. Topographs of areas studied in **a** and **b**, respectively. Source data

To visualize the QSB dispersion **k**(*E*) of UTe_2_ we next use superconductive tip $a\left({\bf{r}},V\right):$ $a\left({\bf{q}},V\right)$ measurements to image energy resolved QPI at the (0–11) cleave surface. Figure 4a–f presents the measured $a({\bf{r}},V)$ at *V* = 0, 50, 100, 150, 200 and 250 µV recorded at *T* = 280 mK in the identical FOV as in Fig. 3a. These data are highly typical of such experiments in UTe_2_. Figure 4h contains the consequent scattering interference patterns $a({\bf{q}},V)$ at *V* = 0, 50, 100, 150, 200 and 250 µV as derived by Fourier analysis of Fig. 4a–f. Here the energy evolution of scattering interference of the QSB states is manifest. For comparison with theory, detailed predicted characteristics of $N\left({\bf{q}},E\right)$ for a *B*_2__*u*_ QSB and a *B*_3__*u*_ QSB at the (0–11) SBZ are presented in Fig. 4g,i; here again, energies are *E* = 0, 50, 100, 150, 200 and 250 µeV. Each QPI wavevector is determined by maxima in the $N\left({\bf{q}},E\right)$ QPI pattern (coloured circles in Fig. 4g–i); these phenomena are highly repeatable in multiple independent experiments (Methods and Extended Data Fig. 10). The general correspondence of *B*_3__*u*_*-*QSB theory to the experimental QPI data is striking. Notably, the strongly enhanced QPI features occurring along the arc connecting ${\bf{q}}=0$ and ${{\bf{q}}}_{5}$ (Fig. 4h) are characteristic of the theory for a *B*_3__*u*_ QSB (Fig. 4i). The arc connecting ${{\bf{q}}}_{1}$ and ${{\bf{q}}}_{2}$ (Fig. 4i) is the consequence of projected FS scattering, and it is irrelevant to the superconducting order parameter $\Delta ({\bf{k}})$. Most critically, however, the intense QPI appearing at wavevector ${{\bf{q}}}_{1}$ (red circles in Fig. 4h,i) is a characteristic of the *B*_3__*u*_ superconducting state, deriving from its geometrically unique nodal structure (Extended Data Fig. 5). Further analysis involving the calculation of the spin-resolved surface spectral function (Extended Data Fig. 4) establishes that scattering at ${{\bf{q}}}_{1}$ is suppressed for *B*_2__*u*_ gap symmetry due to proscribed spin-flip scattering processes but is uniquely enhanced for *B*_3__*u*_ gap symmetry. Moreover, the appearance of scattering interference of QSB quasiparticles at **q**_1_ in the superconducting state (Fig. 3d) is as anticipated by theory^19,31^ due to projection of *B*_3__*u*_ energy-gap nodes on the bulk FS (Fig. 2) onto the (0–11) crystal surface 2D BZ.

Although the superconductive QSB of UTe_2_ has now been rendered directly accessible to visualization (Figs. 3 and 4), its precise topological categorization^19,20,23–34^ depends on details of the normal-state FS that have not yet been determined conclusively^10,11^. Nevertheless, major advances in empirical knowledge of both the QSB and the bulk ${\Delta }_{{\bf{k}}}$ symmetry of the putative topological superconductor UTe_2_ have been achieved. By introducing superconductive scan-tip Andreev tunnelling spectroscopy, which is specifically sensitive to the QSB of ITSs, we visualize dispersive QSB scattering interference of UTe_2_ (Figs. 3d and 4h). This reveals exceptional in-gap QPI patterns exhibiting a characteristic sextet of wavevectors **q**_*i*_: *i* = 1–6 (Fig. 3d,e) that we demonstrate are due to projection of the bulk superconductive band structure (Fig. 1a,b), mathematically equivalent to a rotation making the point of view perpendicular to the (0–11) plane (Fig. 1f). Thence, we find that, whereas ${{\bf{q}}}_{2}$ and ${{\bf{q}}}_{6}$ are weakly observable in the normal state and ${{\bf{q}}}_{4}$ is a Bragg peak of the (0–11) surface, features at ${{\bf{q}}}_{5}$ and ${{\bf{q}}}_{6}$ become strongly enhanced for superconducting state QPI at $\left|E\right| < \Delta$ (Fig. 3e). Most critically, intense QPI appears at wavevector ${{\bf{q}}}_{1}$ uniquely in the superconducting state (Figs. 3e and 4h). This complete QSB phenomenology (Figs. 3d,e and 4h) is, by correspondence with theory (Figs. 1, 2 and 4g,i), most consistent with a *B*_3__*u*_ symmetry superconducting order parameter. Collectively, we identify the *B*_3__*u*_ state in particular: first, because its unique nodal structure enhances the spectral weight of the QSB responsible for the arc-like feature connected to ${{\bf{q}}}_{5}$ in the superconducting state (Fig. 4h) and, second, because *B*_3__*u*_ is the only state that produces intense QPI at wavevector ${{\bf{q}}}_{1}$ uniquely in the superconducting state (red circle in Fig. 4h,i).

These considerations indicate that UTe_2_ sustains a three-dimensional (3D), odd-parity, spin-triplet, time-reversal-symmetry-conserving, **a**-axis nodal superconducting order parameter (Fig. 2). Moreover, we establish how this 3D ${\Delta }_{{\bf{k}}}$ on its host FS is projected onto the 2D SBZ, generating a superconductive in-gap QSB (Fig. 3d) consistent with general theory for ITSs^21,22^ and other related results^47^. Overall, the data indicate that the superconductive QSB QPI phenomenology (Fig. 4) is a direct consequence of the **k**-space geometry of the FS projected onto the crystal surface of UTe_2_, reveals the existence and energy dispersion ${{\bf{k}}}_{\sigma }\left(E\right)$ of this exceptional in-gap QSB and provides prefatorial evidence that its quasiparticle scattering interference is due to *B*_3__*u*_-symmetry bulk superconductivity in UTe_2_. Most generally, the techniques initiated here represent a particularly promising approach for the identification of ITSs.

## Methods

### UTe_2_ normal-state electronic structure model

In this section, we first consider a four-band tight-binding model reproducing the quasirectangular FS of UTe_2_ and its undulations along ${k}_{z}$ axis, as outlined in ref. ^48^. The characteristic features are assumed to arise from the hybridization between two quasi-one-dimensional chains: one originating from the Te(2) 5*p* orbitals and the other from the U 6*d* orbitals. The lattice constants are taken to be $a=0.41$ nm, $b=0.61$ nm and $c=1.39$ nm.

The coupling between the two Uranium orbitals is modelled by the following Hamiltonian:

6 $$\begin{array}{l}{H}_{{\rm{U}}-{\rm{U}}}=\\\left[\begin{matrix}{\mu }_{\rm{U}}-{2t}_{\rm{U}}\cos {k}_{x}a-{2t}_{{\rm{ch}},\rm{U}}\cos {k}_{y}b &-{\Delta}_{\rm{U}}-2{t}_{\rm{U}}^{{\prime} }\cos {k}_{x}a-2{t}^{\prime}_{{\rm{ch}},\rm{U}}\cos {k}_{y}b-4{t}_{z,\rm{U}}{e}^{-i{k}_{z}c/2}\cos {k}_{x}\frac{a}{2}\cos {k}_{y}\frac{b}{2}\\ -{\Delta}_{\rm{U}}-2{t}_{U}^{{\prime} }\cos {k}_{x}a-2{t}^{\prime}_{{\rm{ch}},\rm{U}}\cos {k}_{y}b -4{t}_{z,\rm{U}}{e}^{i{k}_{z}c/2}\cos {k}_{x}\frac{a}{2}\cos {k}_{y}\frac{b}{2} & {\mu }_{\rm{U}}-{2t}_{\rm{U}}\cos {k}_{x}a-{2t}_{{\rm{ch}},\rm{U}}\cos {k}_{y}b\end{matrix}\right]\end{array}$$

Here the tight-binding parameters are the chemical potential ${\mu }_{U}$, the intradimer overlap ${\Delta}_{U}$ of the uranium dimers (where two uranium atoms are coupled along the **c** axis and the dimers run along the **a** axis), the hopping ${2t}_{U}$ along the uranium chain in the *a* direction, the hopping ${t}_{U}^{{\prime} }$ to other uranium in the dimer along the chain direction, the hoppings *t*_ch,U_ and *t*′_ch,U_ between chains in the *a–b* plane and the hopping *t*_*z*,U_ between chains along the **c** axis.

Similarly, the coupling between the two tellurium orbitals is given by

7 $$\begin{array}{l}{H}_{{\rm{Te}}-{\rm{Te}}}=\\\left[\begin{matrix}{\mu }_{{\rm{Te}}}-{2t}_{{\rm{ch}},{\rm{Te}}}\cos {k}_{x}a &-{\Delta}_{{\rm{Te}}}-{t}_{{\rm{Te}}}{e}^{-i{k}_{y}b}-2{t}_{z,{\rm{Te}}}\cos {k}_{z}\frac{c}{2}\cos {k}_{x}\frac{a}{2}\cos {k}_{y}\frac{b}{2}\\-{\Delta}_{{\rm{Te}}}-{t}_{{\rm{Te}}}{e}^{i{k}_{y}b}-2{t}_{z,{\rm{Te}}}\cos {k}_{z}\frac{c}{2}\cos {k}_{x}\frac{a}{2}\cos {k}_{y}\frac{b}{2}&{\mu }_{{\rm{Te}}}-{2t}_{{\rm{ch}},{\rm{Te}}}\cos {k}_{x}a\end{matrix}\right]\end{array}$$

where the Te tight-binding parameters are the chemical potential *μ*_Te_, the intra-unit-cell overlap ∆_Te_ between the two Te(2) atoms along the chain direction, the hopping *t*_Te_ along the Te(2) chain in the *b* direction, the hopping *t*_ch,Te_ between chains in the *a* direction and the hopping *t*_*z*,Te_ between chains along the *c* axis.

The hybridization between the uranium and tellurium orbitals is given by

8 $${H}_{\rm{U}-{\rm{Te}}}=\left(\begin{array}{cc}\delta & 0\\ 0 & \delta \end{array}\right)$$

The normal-state tight-binding Hamiltonian of UTe_2_ can thus be written as

9 $${H}_{{\rm{UTe}}_{2}}=\left(\begin{array}{cc}{H}_{\rm{U}-\rm{U}} & {H}_{\rm{U}-{\rm{Te}}}\\ {H}_{\rm{U}-{\rm{Te}}}^{+} & {H}_{{\rm{Te}}-{\rm{Te}}}\end{array}\right)$$

We consider the following values for the tight-binding parameters (all parameter values are expressed in units of eV): $${\mu }_{\rm{U}}=-0.355,{\Delta}_{\rm{U}}$$$$=0.38,{t}_{\rm{U}}=0.17,$$$${t}_{\rm{U}}^{{\prime}}=0.08,{t}_{{\rm{ch}},\rm{U}}$$$$=0.015,{t}_{{\rm{ch}},\rm{U}}^{{\prime} }=0.01,$$$${t}_{z,\rm{U}}$$$$=-0.0375,{\mu}_{{\rm{Te}}}=-2.25,$$$${\Delta}_{{\rm{Te}}}$$$$=-1.4,{t}_{{\rm{Te}}}=-1.5,0,$$$${t}_{{\rm{ch}},{Te}}=0,{t}_{z,{\rm{Te}}}$$$$=-0.05,\delta =0.13$$. These parameters are chosen to be consistent with both quantum oscillation measurements and our QPI data. All the hopping terms considered here are between the two nearest neighbours such that all scattering will be constrained to nearest neighbour sites at the surface. Any impurity potential is taken to be fully diagonal in the orbital basis with equal intensity on U orbitals and Te orbitals. These parameters are used in all simulations presented herein.

### UTe_2_ superconductive energy-gap nodes and their (0–11) projections

Nodal locations presented in the main text are derived from the general expression for the electronic dispersion of a spin-triplet superconductor^6^

10 $${E}_{{\bf{k}}}^{\pm }=\sqrt{{\varepsilon }^{2}\left({\bf{k}}\right)+{|{\bf{d}}\left({\bf{k}}\right)|}^{2}\pm |{\bf{d}}({\bf{k}})\times {{\bf{d}}}^{* }({\bf{k}})|}$$

where $\varepsilon \left({\bf{k}}\right)$ is the normal-state dispersion measured from the chemical potential and **d**(**k**) is the **d**-vector order parameter. The gap functions we have considered are those associated with the odd-parity irreducible representations (IRs) of the point group *D*_2__*h*_, namely, those presented in Table 3.

In all cases ${\bf{d}}\left({\bf{k}}\right)={{\bf{d}}}^{* }({\bf{k}})$, the gap function is unitary and the nodal locations are defined by FS intersections with the high-symmetry lines of the BZ. Within this model, the nodal points are indicated by yellow dots in Extended Data Fig. 1a–c for ${B}_{1u}$, ${B}_{2u}$ and ${B}_{3u}$, respectively. For ${B}_{1u}$ symmetry, the FS is fully gapped. Although sharing the same number of independent nodes, the locations of the nodes are extremely different in the 3D Brillion zone for the ${B}_{2u}$ and ${B}_{3u}$ order parameters (Extended Data Fig. 1b,c).

Next, we project the normal-state FS onto the (0–11) plane oriented at an angle of 24° between the normal to the (0–11) plane and the crystal **b** axis (Extended Data Fig. 1d). The result is a (0–11) SBZ. The basis vectors on this (0–11) plane are ${{\bf{e}}}_{a}=\left(\mathrm{1,0,0}\right)$ and ${{\bf{e}}}_{{c}^{* }}=\left(0,\sin \theta ,\cos \theta \right)$, where $\theta =24^\circ$. When an arbitrary vector of (*a*,*b*,*c*) is projected to the (0–11) plane, the projected vector is $\left(\left(a,b,c\right)\cdot {{\bf{e}}}_{a},\left(a,b,c\right)\cdot {{\bf{e}}}_{{c}^{* }}\right)=(a,0.4b+0.91c)$. This occurs because any momentum **k** of the bulk BZ can be decomposed into momentum components parallel to the plane **k**_**||**_ and components perpendicular to the plane **k**_**⊥**_ of the surface. Then only **k**_**||**_ will contribute to the surface quasiparticle states, as **k**_**⊥**_ is no longer a conserved quantity; that is, the (001) quasiparticle states that are transformed into **k**_**⊥**_ states in the (0–11) plane no longer contribute. This is why the scale of **q** space and the size of the SBZ are both reduced when viewed at the (0–11) termination surface of UTe_2_.

Finally, we project the bulk nodes onto the (0–11) plane and obtain a **k**-space projected-nodal structure for order parameters ${B}_{1u}$, ${B}_{2u}$ and ${B}_{3u}$, respectively (Extended Data Fig. 1e–g). By definition, ${A}_{u}$ and ${B}_{1u}$ have no bulk or projected energy-gap nodes, so we consider them no further. However, at the (0–11) SBZ of UTe_2_, the projected-nodal locations of the bulk ${B}_{2u}$ order parameter are fundamentally different from those of the bulk ${B}_{3u}$ order parameter, as shown in Extended Data Fig. 1f,g, respectively.

### Quasiparticle scattering interference in the QSB at the (0–11) surface of UTe_2_

We choose to work in the following basis, where U_1/2_ and Te_1/2_ denote, respectively, the two uranium and tellurium orbitals:

11 $${{\rm{\psi }}}^{+}\left({\bf{k}}\right)=({c}_{{U}_{1},{\bf{k}},\sigma }^{+},{c}_{{U}_{2},{\bf{k}},\sigma }^{+},{c}_{T{e}_{1},{\bf{k}},\sigma }^{+},{c}_{T{e}_{2},{\bf{k}},\sigma }^{+},{c}_{{U}_{1},-{\bf{k}},\bar{\sigma }},{c}_{{U}_{2},-{\bf{k}},\bar{\sigma }},{c}_{T{e}_{1},-{\bf{k}},\bar{\sigma }},{c}_{T{e}_{2},-{\bf{k}},\bar{\sigma }})$$

12 $${c}_{\alpha ,{\bf{k}},\sigma }^{+}=({c}_{\alpha ,{\bf{k}},\uparrow }^{+},{c}_{\alpha ,{\bf{k}},\downarrow }^{+})$$

13 $${c}_{\alpha ,{\bf{k}},\bar{{\boldsymbol{\sigma }}}}=({c}_{\alpha ,{\bf{k}},\downarrow },{c}_{\alpha ,{\bf{k}},\downarrow })$$

In this basis, the BdG Hamiltonian of a *p*-wave spin-triplet superconductor can be written as

14 $${H}_{{\rm{BdG}}}\left({\bf{k}}\right)={{\rm{\psi }}}^{+}\left({\bf{k}}\right)\left(\begin{array}{ll}{H}_{{\rm{UT}}{{\rm{e}}}_{2}}\left({\bf{k}}\right)\bigotimes {I}_{2}\qquad\Delta \left({\bf{k}}\right)\bigotimes {I}_{4}\\ {\Delta }^{+}\left({\bf{k}}\right)\bigotimes {I}_{4}\qquad\;-{H}_{{\rm{UT}}{{\rm{e}}}_{2}}^{* }\left(-{\bf{k}}\right)\bigotimes {I}_{2}\end{array}\right){\rm{\psi }}({\bf{k}})$$

where the order parameter for the putative *p*-wave superconductor is $\Delta \left({\bf{k}}\right)={\Delta }_{0}i\left({\bf{d}}\cdot {\bf{\upsigma }}\right){\sigma }_{2}$, and *I*_n_ is an *n* × *n* identity matrix. In our analysis, we focus on the non-chiral order parameters: ${A}_{u}$, ${B}_{1u}$, ${B}_{2u}$ and ${B}_{3u}$. The **d** vectors used in calculations for each IR are provided in Table 3.

**Table 3:** Odd-parity irreducible representations of the crystal point symmetry group *D*_2__*h*_ and corresponding **d** vectors representations for the simple orthorhombic lattice model used throughout this Article

IR	d vector
*A*_*u*_	$[{{\rm{C}}}_{1}\sin \left({k}_{x}a\right),{C}_{2}\sin \left({k}_{y}b\right),{C}_{3}\sin \left({k}_{z}c\right)]$
*B*_1__*u*_	${[C}_{1}\sin \left({k}_{y}b\right),{C}_{2}\sin \left({k}_{x}a\right),{C}_{0}\sin {\rm{}}({k}_{x}a)\sin {\rm{}}({k}_{y}b)\sin {\rm{}}({k}_{z}c)]$
*B*_2__*u*_	$[{C}_{1}\sin \left({k}_{z}c\right),{C}_{0}\sin \left({k}_{x}a\right)\sin \left({k}_{y}b\right)\sin \left({k}_{z}c\right),{C}_{3}\sin {\rm{}}({k}_{x}a)]$
*B*_3__*u*_	$[{C}_{0}\sin \left({k}_{x}a\right)\sin \left({k}_{y}b\right)\sin \left({k}_{z}c\right),{C}_{2}\sin \left({k}_{z}c\right),{C}_{3}\sin {\rm{}}({k}_{y}b)]$

In our simulations, we hypothesize the following values: *C*_0_ = 0, *C*_1_ = 300 µeV, *C*_2_ = 300 µeV and *C*_3_ = 300 µeV. In this conventional model ${C}_{1}$, ${C}_{2}$ and ${C}_{3}$ are hypothesized to be the same as the UTe_2_ gap amplitude measured in the experiment. Although the relative intensity of these coefficients is not known a priori, we have checked that, while keeping the maximum gap constant, these coefficient values produce the same QPI features with only slight changes in wavevector length. Within this model, the unperturbed retarded bulk 3D Green’s function is given as

15 $${G}_{0}({\boldsymbol{k}},\omega )={[(\omega +i{\eta })I-{H}_{{\rm{BdG}}}({\boldsymbol{k}})]}^{-1}$$

with the corresponding unperturbed spectral function written as

16 $${A}_{0}({\boldsymbol{k}},\omega )=-1/\pi {Im}{G}_{0}({\boldsymbol{k}},\omega )$$

where *η* is the energy-broadening factor in the theory simulation.

Although obtaining the bulk Green’s function is straightforward, calculating the surface Green’s functions and spectral functions *A*_s_(**k**,ω) is substantially more difficult. The complexity arises because the surface Green’s functions characterize a semi-infinite system with broken translational symmetry, and thus they cannot be calculated directly. Traditionally, they are obtained using heavy numerical recursive Green’s function techniques as in ref. ^49^. Here we use a simpler analytical technique, described in refs. ^44–46^, in which the surface is modelled using a planar impurity. When the magnitude of the impurity potential goes to infinity, the impurity splits the system into two semi-infinite spaces. Then only wavevectors in the (0–11) plane remain good quantum numbers. The effect of this impurity can be exactly calculated using the T-matrix formalism, which gives one access to the surface Green’s function of the semi-infinite system.

We model the effect of the surface using a planar-impurity potential, as in Extended Data Fig. 2, which is oriented parallel to the (0–11) crystal plane. In the presence of this impurity, the bulk Green’s function is modified to

17 $$G\left({{\bf{k}}}_{1},{{\bf{k}}}_{2},\omega \right)={G}_{0}\left({{\bf{k}}}_{1},\omega \right){\delta }_{{{\bf{k}}}_{1},{{\bf{k}}}_{2}}+{G}_{0}\left({{\bf{k}}}_{1},\omega \right)T\left({{\bf{k}}}_{1},{{\bf{k}}}_{2},\omega \right){G}_{0}\left({{\bf{k}}}_{2},\omega \right)$$

where the T matrix considers all-order impurity scattering processes. For a plane impurity localized at $x=0$ and perpendicular to the *x* axis, the T matrix can be computed as

18 $$T\left({k}_{1y},{k}_{1z},{k}_{2y},{k}_{2z},\omega \right)={\delta }_{{k}_{1y},{k}_{2y}}{\delta }_{{k}_{1z},{k}_{2z}}[1-\hat{V}\int \frac{d{k}_{x}}{{L}_{x}}{G}_{0}\left({k}_{x},{k}_{1y},{k}_{1z},\omega \right)]^{-1}\hat{V}$$

with ${L}_{x}$ a normalization factor. Because the impurity potential is a delta function in *x*, the T matrix is independent of ${k}_{x}$ and depends only on ${k}_{y}$ and ${k}_{z}$.

We calculate the exact Green’s function one lattice spacing away from the planar-impurity potential, which converges precisely to the surface Green’s function as the impurity potential approaches infinity. This surface Green’s function can be obtained by performing a partial Fourier transform of the exact Green’s function expressed in equation (17):

19 $${G}_{s}\left({k}_{y},{k}_{z}\right)=\int \frac{d{k}_{1x}}{{L}_{x}}\int \frac{d{k}_{2x}}{{L}_{x}}G\left({k}_{1x},{k}_{y},{k}_{z},{k}_{2x},{k}_{y},{k}_{z},\omega \right){e}^{i{k}_{1x}x}{e}^{-i{k}_{2x}{x}^{{\prime} }}$$

where $$x={x}^{{\prime} }=\pm 1$$.

Extended Data Fig. 3a–c is generated using the above (0–11) planar-impurity-potential formalism for the four-band model with *B*_1__*u*_, *B*_2__*u*_ and *B*_3__*u*_ gap structures. In Extended Data Fig. 3, we present the surface spectral function *A*_s_(**k**, *E*) for these order parameters in the (0–11) SBZ. In particular, the surface spectral function *A*_s_(**k**, *E*) for *B*_3__*u*_ in the (0–11) SBZ is shown in Extended Data Fig. 3c. A hypothesized sextet of scattering wavevectors **q**_*i*_, *i* = 1–6 connecting regions of maximum intensity in *A*_s_(**k**, 0) is overlaid. All plots show data for six energy levels, with the highest near the gap edge of $|{\Delta}_{\text{UT}{{\rm{e}}}_{2}}|=300$ µeV.

We next describe how QPI scattering is possible given the putative protection of superconductive topological surface band quasiparticles against scattering in a topological superconductor. Formally, we can derive the spin-resolved quasiparticle surface spectral function as shown for a *B*_2__*u*_ and *B*_3__*u*_ QSB in Extended Data Fig. 4. The resulting surface spectral function can be clearly segregated into two spin-polarized bands in UTe_2_, one for each spin eigenstate. Although spin-flip and thus inter-spin-band scattering is proscribed, non-spin-flip or intra-spin-band scattering is allowed, thus allowing QPI of these quasiparticles.

Extended Data Fig. 5a,b depicts the projection of the bulk spectral function of order parameters ${B}_{2u}$ and ${B}_{3u}$ on the (0–11) surface. It should be noted that the resulting features correspond to regions identifiable from the 3D bulk FS as the projection of the bulk nodes onto the (0–11) surface, and these features are highlighted by yellow circles. Extended Data Fig. 5c,d depicts the surface spectral function *A*_s_(**k**, 0) computed using the planar-impurity method^44^. It accounts for some bulk contributions but is dominated by new features that connect the projection of the bulk nodes to the SBZ; these new features correspond to the QSB of order parameters ${B}_{2u}$ and ${B}_{3u}$.

In Extended Data Fig. 5e,f, we consider (0–11) surface QPI featuring order parameters of ${B}_{2u}$ and ${B}_{3u}$ symmetry using the JDOS $J\left({\boldsymbol{q}},0\right)$. The JDOS approximation $J\left({\bf{q}},0\right)$ is a well-established technique to map out the geometries of the momentum-space band structures^36^. The JDOS approximation is based on the observation that if the surface spectral functions ${A}_{{\rm{s}}}$ at **k** and **k** + **q** are both simultaneously large, then $J\left({\bf{q}},E\right)$ will be large, as **q** connects regions of large JDOS. This technique has been used to successfully interpret the experimental QPI data for high-temperature superconductors^50,51^, topological insulators^52,53^ and Weyl semimetals^54,55^.

Although $J\left({\bf{q}},E\right)$ captures the dominant **k**-space quasiparticle scattering associated with the order-parameter symmetries, it does not consider spin-forbidden scattering processes and the underlying contributions from the bulk band structure as accurately as the $N\left({\bf{q}},E\right)$ simulations presented in the main text. However, both $J\left({\bf{q}},E\right)$ and $N\left({\bf{q}},E\right)$ calculations reveal distinct scattering features.

We show the theoretical *N(E)* calculations for the UTe_2_ (0–11) surface with *B*_2__*u*_ and *B*_3__*u*_ gap symmetries in Extended Data Fig. 5g,h. Both gap symmetries show the indistinguishable bulk *N(E)* of a nodal *p*-wave superconductor (black curve). The *N(E)* at the surface (red curve) differs entirely between the two order-parameter symmetries in this model. For a *B*_3__*u*_ order parameter, the surface *N(E)* has a clear zero-energy peak; however, the surface *N(E)* due to a *B*_2__*u*_ order parameter has only reduced gap depth compared to bulk. In the experiment, we find intense zero-energy conductance, which appears most consistent with the (0–11) surface *N(E)* in the presence of *B*_3__*u*_ gap symmetry.

To further improve the comparison between the QPI simulations and the experimental QPI data, we consider of the **q**-space sensitivity of our scan tip in the QPI simulations. The QPI simulations $N\left({\bf{q}},E\right)$ for the *B*_2__*u*_ and *B*_3__*u*_ order parameters are shown in Extended Data Fig. 6a,b, which shows very strong intensities near the high-**q** region. In experimental data, however, the intensity near the high-**q** regions that represent the shortest distances in **r** space decays rapidly due to the finite radius of the scan tip. We estimate the actual **q**-space intensity decay radius from a Gaussian fit to the power spectral density of the relevant *T*(**q**) image. Subsequently, we apply a 2D Gaussian function of the following form to the QPI simulations $N\left({\bf{q}},E\right)$, reflecting the effects of the finite circular radius or ‘aperture’ of the scan tip:

20 $$f\left({q}_{x},{q}_{y}\right)=A\exp \left(-\left(\frac{{\left({{\boldsymbol{q}}}_{x}-{{\boldsymbol{q}}}_{{x}_{0}}\right)}^{2}}{2{\sigma }_{x}^{2}}+\frac{{\left({{\boldsymbol{q}}}_{y}-{{\boldsymbol{q}}}_{{y}_{0}}\right)}^{2}}{2{\sigma }_{y}^{2}}\right)\right)$$

where the amplitude $A=1.75\times {10}^{-5}$, the centre coordinates $\left({q}_{{x}_{0}},{q}_{{y}_{0}}\right)=(\mathrm{0,0})$ and the standard deviation ${\sigma }_{x}={\sigma }_{y}=3.68\pi /{c}^{* }$. Upon applying this 2D ‘aperture’ filter in Extended Data Fig. 6a,b, we derive the $N({\bf{q}},E)$ in main text Fig. 4g,i.

To evaluate the effect of impurity strength on the QPI calculations, we performed superconductive topological surface band QPI simulations using local impurity potentials of *V* = 0.07, 0.2, 0.5 and 1 eV potentials and found that the predictions using different scattering impurity potentials lead to highly consistent scattering wavevectors (Extended Data Fig. 6c–f). Varying the scattering potentials only changes relative amplitudes at different wavevectors; when the scattering potentials increase, the scattering wavevectors caused by the surface state become more intense. We chose *V* = 0.2 eV because the QPI simulations calculated using this scattering potential are most consistent with the relative QPI intensities observed experimentally. The scientific conclusion that QPI in the superconductive topological surface state of UTe_2_ is consistent with *B*_3__*u*_ bulk pairing symmetry remains unchanged when using scattering potentials ranging from 0.07 to 1 eV, as presented above.

### SABS in unconventional superconductors

The SABS and concomitant zero-bias conductance peaks due to π phase shifts have been extensively studied for decades, particularly in high-temperature superconductors^17,56–59^. In *d*-wave superconductors such as the cuprates, the π phase shift of the pair potential occurs universally when the angle between the crystal axis of the superconductors and the lobe direction of *d*-wave pair potential is nonzero. This phase shift leads to the formation of SABS due to Andreev reflection. These SABS manifest as zero-bias conductance peaks in tunnelling spectroscopy, a hallmark feature widely observed and investigated in the cuprate high-temperature superconductors.

Although never observed experimentally in a spin triplet superconductor, SABS should emerge in 3D *p*-wave ITSs, where they are often described as superconducting topological surface states. These SABS have a somewhat distinct physical origin from those in *d*-wave systems because in odd-parity superconductors, there is a universal π phase shift of the superconducting order parameter at all surfaces, independent of the angle between the crystal axis and the direction of the phase of the superconducting order parameter.

### Alternative gap function and impurity potential

Owing to the body-centred orthorhombic crystal symmetry of UTe_2_, basis functions other than those presented in the main text and above are allowed. To consider alternative basis functions, we add additional, symmetry-allowed, terms to the **d** vectors as described in ref. ^8^. For the nodal, single-component order parameters, we then use the **d** vectors featured in Table 4 with ${C}_{0}=0$, ${C}_{1}={C}_{2}={C}_{3}=0.225$ $\text{meV}$ and ${C}_{4}={C}_{5}={C}_{6}=0.15$ $\text{meV}$.

**Table 4:** The **d** vector representations for the body-centred orthorhombic lattice model

*B*_2__*u*_	$$\begin{array}{l}\left(\begin{array}{c}{{C}_{1}\sin \left({k}_{z}c\right)+{C}_{4}\sin \frac{{k}_{z}c}{2}\cos \frac{{k}_{x}a}{2}\cos \frac{{k}_{y}b}{2}}\\{C}_{0}\sin ({k}_{x}a)\sin ({k}_{y}b)\sin ({k}_{z}c)\\ {C}_{3}\sin ({k}_{x}a)+{C}_{6}\sin \frac{{k}_{x}a}{2}\cos \frac{{k}_{y}b}{2}\cos \frac{{k}_{z}c}{2}\end{array}\right)\end{array}$$
*B*_3__*u*_	$$\begin{array}{l}\left(\begin{array}{c}{{C}_{0}\sin ({k}_{x}a)\sin ({k}_{y}b)\sin ({k}_{z}c)}\\{C}_{2}\sin ({k}_{z}c)+{C}_{5}\sin \frac{{k}_{z}c}{2}\cos \frac{{k}_{x}a}{2}\cos \frac{{k}_{y}b}{2}\\ {C}_{3}\sin ({k}_{y}b)+{C}_{6}\sin \frac{{k}_{y}b}{2}\cos \frac{{k}_{x}a}{2}\cos \frac{{k}_{z}c}{2}\end{array}\right)\end{array}$$

To establish that the conclusions derived in the main text would be unchanged if these alternative **d** vectors were used, we calculate the bulk projected spectral function *A*_0_(**k**, *E*), surface spectral function *A*_s_(**k**, *E*) and $J\left({\bf{q}},E\right)$ using these alternative triplet **d** vectors. These data are presented in Extended Data Fig. 7 for $E=0$. The nodal pattern highlighted with yellow dashed circles in Extended Data Fig. 7a,b can be directly compared to Extended Data Fig. 5a,b. The alternative **d** vectors have a very similar nodal pattern when projected to the (0–11) plane, and thus the QSBs occupy similar regions of the projected SBZ. This can be seen in Extended Data Fig. 7c,d, in which we plot *A*_*s*_(**k**,0). From comparison with Extended Data Fig. 5c,d, we see clearly that the QSBs calculated using either the main text **d** vector or these alternative **d** vectors are nearly identical. The resulting $J\left({\bf{q}},E\right)$, is presented in Extended Data Fig. 7e,f for order-parameter symmetries *B*_2__*u*_ and *B*_3__*u*_, respectively. Using the same quasiparticle broadening parameter as in Extended Data Fig. 5e,f, $\eta =30$ μeV; but now, with these alternative **d**-vector terms, we see that the $J\left({\bf{q}},E\right)$ QPI patterns predicted for each order parameter have the same key features.

### Andreev conductance *a*(r,*V*) of QSB quasiparticles

A key consideration is the role of QSB-mediated Andreev conductance across the junction between *p*-wave and *s*-wave superconductors (Extended Data Fig. 8). Most simply, a single Andreev reflection transfers two electrons (holes) between the tip and the sample. Based on an S-matrix approach, the formula to compute the Andreev conductance of the *s*-wave–insulator–*p*-wave model is

21 $$a\left(V\;\right)=\frac{8{\pi }^{2}{t}_{{\rm{eff}}}^{\;4}{e}^{2}}{h}\sum _{n}\frac{\left\langle {\phi }_{n}|{P}_{h}|{\phi }_{n}\right\rangle \left\langle {\phi }_{n}|{P}_{e}|{\phi }_{n}\right\rangle }{{\left({eV}-{E}_{n}\right)}^{2}+{\pi }^{2}{t}_{{\rm{eff}}}^{\;4}{\left[\left\langle {\phi }_{n}|{P}_{h}|{\phi }_{n}\right\rangle +\left\langle {\phi }_{n}|{P}_{e}|{\phi }_{n}\right\rangle \right]}^{2}}$$

Here, |${\phi }_{n}\rangle$ is the projection of the *n*th QSB eigenfunction onto the top UTe_2_ surface, ${P}_{e}$ and ${P}_{h}$ are the electron and hole projection operators acting on the UTe_2_ surface, and *V* is the bias voltage. Thus, in principle, and as outlined in ref. ^35^, superconductive scan tips can be employed as direct probes of QSBs, with tip-sample conductance mediated by Andreev transport through the QSB.

### Distinguish between Andreev tunnelling and Josephson tunnelling

Determining whether the physical origin of the zero-bias conductance is due to Josephson or Andreev tunnelling is important. However, Josephson currents are undetectable in all Nb/UTe_2_ junctions that we have studied. This can be demonstrated by comparing the zero-bias (Andreev) conductance *a*(0) versus junction resistance *R* on the same plot with the maximum possible zero-bias conductance *g*(0), which could be generated by the Josephson effect (as shown in Supplementary Fig. 6 of ref. ^35^). First, at high *R*, the intensity of measured *a*(0) of Nb/UTe_2_ junctions is orders of magnitude larger than it could possibly be due to Josephson currents (here exemplified by measured Nb/NbSe_2_ Josephson effect zero-bias conductance^60^ that itself should be at least five times larger than any that could exist in Nb/UTe_2_). Second, measured *a*(0) for Nb/UTe_2_ first grows linearly with falling *R* but then diminishes steeply as *R* is reduced further. By contrast, zero-bias conductance due to Josephson currents *g*(0) must grow rapidly and continuously as 1/*R*^2^, as exemplified in the Nb/NbSe_2_ *g*(0) data^60^. These facts (Supplementary Fig. 6 of ref. ^35^) demonstrate the absolute predominance of Andreev tunnelling and the non-observability of Josephson currents between Nb electrodes and the UTe_2_ (0–11) termination surface.

### Normal-tip and superconductive-tip study of QSBs

Motivated by the presence of dominant finite density of states at zero energy as $T\to 0$ and by the consequent hypothesis that a QSB exists in this material, we searched for its signatures using a non-superconductive tip, at voltages within the superconducting energy gap, and identified unique features resulting from QSB scattering interference. The typical NIS tunnelling conductance of the UTe_2_ superconducting state measured using a non-superconductive tip is exemplified in the inset to Extended Data Fig. 9b. At the (0–11) surface of superconducting UTe_2_ crystals, almost all states inside the superconducting gap ${|E|} < {\Delta }_{0}$ show residual, ungapped density of states. A combination of impurity scattering and the presence of a QSB on this crystal surface are expected for a *p*-wave superconductor. Both types of these unpaired quasiparticles should contribute to conductance measurements performed within the superconducting gap using a non-superconductive scan tip. To visualize the scattering interference of QSB quasiparticles, we focus on a 40-nm-square FOV (Extended Data Fig. 9a) for conventional normal-tip differential conductance ${{\rm{d}}I}/{{\rm{d}}V}{|}_{{\rm{NIS}}}({\bf{r}},V)$ at *T* = 280 mK and at a junction resistance of *R* = 5 MΩ. Although the QPI inside the superconducting gap shows some evidence of the QSB in UTe_2_, its weak signal-to-noise ratio owing to the dominant finite density of states for $\left|E\right|\le {\Delta }_{0}$ implies that conventional d*I/*d*V*|_NIS_ **q**,*V*) spectra are inadequate for precision application of detecting and quantifying the QPI of the QSB in UTe_2_.

Thus, we turned to a new technique by using superconductive tips to increase the signal-to-noise ratio of QSB quasiparticle scattering. Recent theory for the tunnel junction formed between an *s*-wave superconductive scan tip and a *p*-wave superconductor with a QSB within the interface^35^ reveals that the high density of QSB quasiparticles allows efficient creation/annihilation of Cooper pairs in both superconductors, thus generating intense Andreev differential conductance *a*(**r**, *V*) ≡ ${{\rm{d}}I}/{{\rm{d}}V}{\ |}_{{\rm{A}}}({\bf{r}},V)$. This is precisely what is observed when UTe_2_ is studied by superconductive Nb-tip STM at *T* = 280 mK, as evidenced by the large zero-energy conductance peak around *a*(**r**, 0) (inset to Extended Data Fig. 9d). Visualization of *a*(**r**, 0) and its Fourier transform *a*(**q**, 0), as shown in Extended Data Fig. 9d, reveals intense conductance modulations and a distinct QPI pattern. Comparing *g*(**q**, 0) in Extended Data Fig. 9b and *a*(**q**, 0) in Extended Data Fig. 9d reveals numerous common characteristics, thus demonstrating that use of *a*(**q**, *V*) imaging yields equivalent QPI patterns as *g*(**q**, *V*) imaging but with a greatly enhanced signal-to-noise ratio. This is as expected because spatial variations in the intensity of *a*(**r**, *V*) are controlled by the amplitude of QSB quasiparticle wavefunctions as in equation (21), so spatial interference patterns of the QSB quasiparticles will become directly observable in *a*(**r**, *V*). Thus, visualizing spatial variations in *a*(**r**, *V*) and their Fourier transforms *a*(**q**, *V*) enables efficient, high-signal-to-noise-ratio exploration of QSB quasiparticle scattering interference phenomena at the surface of UTe_2_.

### Independent QSB visualization experiments

To confirm that the QPI of the QSB is repeatable, we show two additional examples of the Andreev QPI $a({\bf{q}},0)$ from two different FOVs in Extended Data Fig. 10. The QPI maps $a({\bf{q}},0)$ are measured at zero energy, where the Andreev conductance is most prominent. The two QPI $a({\bf{q}},0)$ maps in Extended Data Fig. 10a,b show vividly the same sextet of scattering wavevectors **q**_*i*_, *i* = 1–6 reported in the main text and further confirm the signatures of a *B*_3__*u*_ QSB in UTe_2_. In particular, repeated measurements of the ${{\bf{q}}}_{1}$ wavevector exclusively both within the superconducting energy gap and at *T* = 280 mK support the presence of a superconducting order parameter with *B*_3__*u*_ symmetry, as this is the only order parameter that allows spin-conserved scattering at ${{\bf{q}}}_{1}$. These two QPI maps are measured independently in two different FOVs and at two different scanning angles (Extended Data Fig. 10c,d).

### Origin of the scattering wavevector ${{\bf{q}}}_{1}$

The interaction with uniform superconductivity of the UTe_2_ pre-existing charge density wave (CDW) or of the consequent pair density wave (PDW), both occurring with the same wavevector **Q** = **q**_6_, cannot induce either a CDW or a PDW at **Q**/2. This is ruled out by Ginzburg–Landau theory^61^. As to the appearance of a new fundamental PDW at a **q**_1_, this has been ruled out previously by direct search for energy-gap modulations at that wavevector^13^.

The emergence of **q**_1_ scattering intensely in the superconducting state of UTe_2_ occurs naturally because this wavevector arises from Bogoliubov quasiparticle scattering between symmetry-imposed superconducting nodes of the *B*_3__*u*_ order parameter^34^. In the normal state, scattering between FSs at this wavevector may also occur, but it is not predominant.

Notably, the superconducting gap nodes of the *B*_3__*u*_ order parameter coincide with the location of the normal-state FS nesting points. Consequently, the QPI wavevectors observed in the superconducting state of UTe_2_ (Fig. 3d) coincide with the normal-state FS nesting vectors. This is not necessarily the case in other superconductors such as Sr_2_RuO_4_, where the Bogoliubov QPI scattering wavevectors are entirely different from the normal-state FS nesting vectors because of the locations of the nodes in that material^43^.

## Online content

Any methods, additional references, Nature Portfolio reporting summaries, source data, extended data, supplementary information, acknowledgements, peer review information; details of author contributions and competing interests; and statements of data and code availability are available at 10.1038/s41567-025-03000-w.

## Source data

Source Data Fig. 1 Source data for Fig. 1a–f.

Source Data Fig. 2 Source data for Fig. 2a–b.

Source Data Fig. 3 Source data for Fig. 3a–e.

Source Data Fig. 4 Source data for Fig. 4a–i.

Source Data Extended Data Fig. 1 Source data for Extended Data Fig. 1.

Source Data Extended Data Fig. 3 Source data for Extended Data Fig. 3a–c.

Source Data Extended Data Fig. 4 Source data for Extended Data Fig. 4a–b.

Source Data Extended Data Fig. 5 Source data for Extended Data Fig. 5a–h.

Source Data Extended Data Fig. 6 Source data for Extended Data Fig. 6a–f.

Source Data Extended Data Fig. 7 Source data for Extended Data Fig. 7a–f.

Source Data Extended Data Fig. 8 Source data for Extended Data Fig. 8b.

Source Data Extended Data Fig. 9 Source data for Extended Data Fig. 9a–d.

Source Data Extended Data Fig. 10 Source data for Extended Data Fig. 10a–d.

## Data Availability

The source data shown in the main figures and Extended Data figures are available from Zenodo via 10.5281/zenodo.15597299 (ref. ^62^). Source data are provided with this paper.

## Code availability

The source code used to perform the calculations described in this paper is available from the corresponding authors upon request.
